# Advanced nonlinear dynamics and bifurcation structures in multi-coupled oscillators using a powerful non-perturbative framework

**DOI:** 10.1038/s41598-026-44027-0

**Published:** 2026-04-07

**Authors:** Galal M. Moatimid, Yasmeen M. Mohamed, M. K. Abohamer

**Affiliations:** 1 Department of Mathematics, Faculty of Education, Ain Shams University, Cairo, 11566 Egypt; 2 Department of Engineering Physics and Mathematics, Faculty of Engineering, Tanta University, Tanta, Egypt

**Keywords:** Multi-degree-of-freedom nonlinear oscillators, Analytical–numerical approach, Strongly nonlinear dynamics, Bifurcation diagrams, Poincaré maps, Engineering, Mathematics and computing, Physics

## Abstract

Nonlinear oscillators with two degrees of freedom (2DOF) serve as fundamental models for describing complex dynamical behavior in engineering and applied mechanics. Accurate prediction of their responses is crucial for stability enhancement, vibration suppression, and optimal design of coupled mechanical systems. In this study, three distinct 2DOF coupled oscillator models are examined, encompassing both linear and strongly nonlinear restoring forces that govern free and damped vibration regimes. These models provide realistic frameworks for analyzing nonlinear interactions, resonance phenomena, and stability boundaries in coupled dynamical systems. The primary objective is to develop and apply a robust non-perturbative approach (NPA) for deriving periodic solutions of conservative and damped coupled oscillators. The proposed approach, rooted in He’s Frequency Formula (HFF), fundamentally differs from classical perturbation techniques as it avoids Taylor-series expansions, linearization assumptions, and small-parameter constraints. Instead, the nonlinear governing equations are transformed into analytically tractable linear forms, enabling efficient treatment of strongly nonlinear performance. The analytical solutions are validated through comprehensive numerical simulations implemented in Mathematica Software (MS), and are systematically compared with direct numerical integrations, demonstrating excellent accuracy and computational efficiency. Furthermore, bifurcation diagrams and Poincaré maps (PMs) are employed to characterize the qualitative dynamical transitions and classify the complex response patterns exhibited by each coupled model.

## Introduction

Dynamical systems with 2DOF organize fundamental, essentially complex models in nonlinear dynamics, offering deep mathematical and physical insight into a wide spectrum of engineering and applied mechanics problems. Owing to their ability to capture modal interactions, internal resonances, and nonlinear energy exchange mechanisms. Such systems were extensively studied under diverse successful conditions, including external harmonic excitation^1^, viscous damping effects, and nonlinear stiffness characteristics. Analytical approaches, most notably the multiple time-scales method^2^, were widely employed to explore resonance phenomena, stability properties, and nonlinear modal interactions, while fractional-order formulations were introduced to improve the accuracy of harmonic balance-based solutions^3^. Additional studies addressed 2DOF oscillators evolving in potential fields, elucidating the influence of conformal coordinates and gyroscopic effects^4^, as well as escape dynamics from multi-well potentials under resonant and non-resonant excitations^5^. Investigations of collective behavior in coupled van der Pol–Duffing oscillators with inertial and dissipative coupling^6^, analyses of dissipation-induced chaos in coupled quartic oscillators^7^, and pendulum-based 2DOF models with applications in high-precision inertial sensing^8^ further demonstrate the broad relevance of such systems. Despite these substantial advances, the analytical treatment of coupled nonlinear oscillators remains challenging, particularly when strong nonlinearities and complex coupling mechanisms invalidate conventional simplifying assumptions. The existing study distinguishes itself from previous research in its breadth and methodology. The work presents a novel framework, utilizes alternative analytical methods, and investigates fresh aspects of the topic, in contrast to previous studies that concentrated on comparable themes or adhered to conventional procedures. Consequently, it provides novel insights and makes a distinctive contribution to the existing body of knowledge.

There has been sustained interest in nonlinear Duffing oscillators (Dos) due to their effectiveness in modeling a wide range of nonlinear behaviors encountered in engineering systems. Among the analytical tools developed for such problems, perturbation-based techniques have played a dominant role. The homotopy perturbation method (HPM), often combined with modified Lindstedt–Poincaré procedures, was applied to analyze nonlinear free vibrations of beams under various boundary and loading conditions^9^. Through Galerkin reduction, complex continuous systems were transformed into reduced-order nonlinear ordinary differential equations (ODEs), enabling approximate analytical treatments of cubic–quintic DOs and Duffing–harmonic oscillators with reasonable accuracy^10^. Considerable efforts were devoted to enhancing the precision of perturbation-based solutions^11^, including studies on dynamic pull-in instability in nano-torsional switches^12^ and the application of global residue harmonic balance to nano-electromechanical resonators^13^. Variational iteration methods have also been refined through systematic improvements in the correction functional employing Lagrange multipliers^14^. Furthermore, hybrid approaches combining HPM with classical perturbation techniques were proposed to mitigate certain limitations associated with small-parameter assumptions^15^. Alternative perturbation strategies have demonstrated improved performance in selected strongly nonlinear regimes^16^. More recently, auto-parametric 2DOF systems subjected to external torque and periodic excitation were investigated using the multiple time-scales method, yielding approximate analytical solutions and insight into their nonlinear dynamic evolution^17^. Nevertheless, the accuracy and robustness of perturbation-based formulations often deteriorate in the presence of strong nonlinearities, non-conservative effects, or complex coupling interactions. This study differentiates itself from prior works through its scope and methods. The study introduces an innovative framework, employs alternative analytical techniques, and explores new dimensions of the subject, unlike prior research that focused on similar subjects or followed traditional methodologies. Thus, it offers innovative perspectives and makes a unique contribution to the current body of knowledge.

Nonlinear instability is a fundamental concept in the analysis and control of complex dynamical behavior observed in many physical and engineering systems. In particular, nonlinear vibrations play a central role in mechanical and electrical applications where strong nonlinearities and coupling effects govern the system response. Within this context, dynamical oscillators serve as canonical models in investigating nonlinear response characteristics under various excitations and damping conditions. Among NPA tools, HFF has demonstrated notable efficiency in predicting frequency-amplitude relationships of nonlinear oscillators without relying on small-parameter expansions or cumbersome series solutions^18–20^. Building upon the core principles of HFF, the NPA has recently emerged as a practical analytical framework in examining nonlinear responses and stability features in diverse dynamical systems, including problems related to Electrohydrodynamics stability. While not intended to replace numerical simulations, NPA provides an efficient analytical complement capable of capturing essential dynamical features of nonlinear oscillators. Motivated by the adamant challenges associated with modeling strongly nonlinear and coupled configurations, the present study employs a modified NPA to investigate the 2DOF of coupled oscillator systems.

The oscillators considered in this work represent canonical and widely accepted models in nonlinear dynamics. Coupled van der Pol oscillators describe systems governed by the interplay between self-excitation, damping, and coupling effects and are commonly employed to analyze organization, bifurcation phenomena, and transitions to chaos. Coupled quartic oscillators with 2DOF serve as prototypical models for strongly nonlinear environments in which higher-order potential terms dominate the system dynamics, enabling the study of nonlinear energy transfer, internal resonance, and strong mode coupling. In contrast, coupled simple harmonic oscillators provide baseline representations of interacting oscillatory units and offer fundamental insight into energy exchange, normal modes, and resonance interactions. The inclusion of these different oscillator classes allows for a systematic assessment of the proposed analytical framework across conservative and dissipative regimes, as well as weakly and strongly nonlinear configurations .

**Fig. 1:**
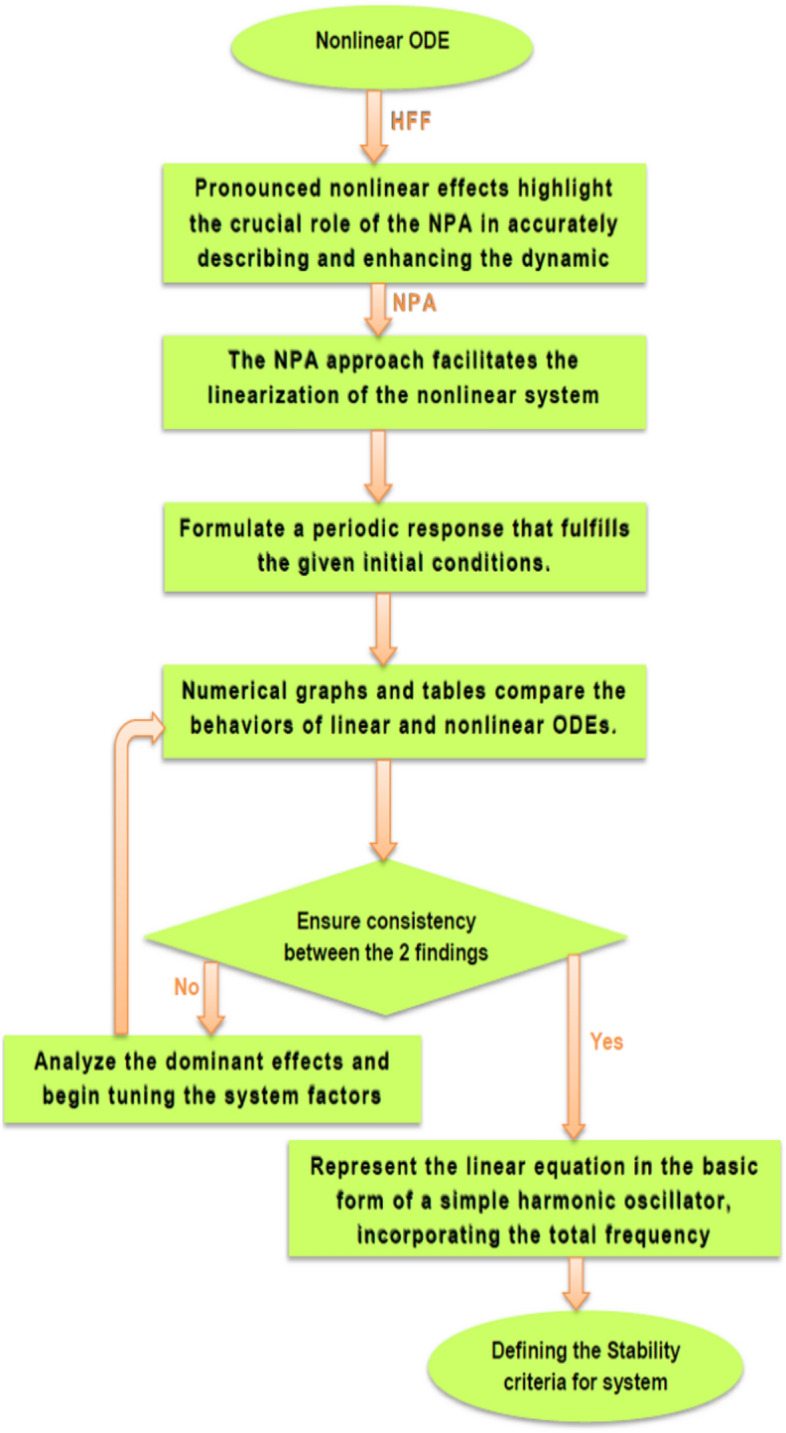
Shows a schematic representation of the proposed methodology, illustrating the sequential steps of the HFF–NPA process.

Despite the extensive body of literature on 2DOF nonlinear oscillators, several critical gaps remain. Most existing investigations rely heavily on perturbation-based analytical techniques that assume weak nonlinearities or the existence of small parameters, which limits their reliability when strong nonlinear impacts, nonlinear damping, or complex coupling mechanisms dominate the system response. Furthermore, diverse studies focus on specific oscillator outlines or isolated dynamical scenarios, rather than providing a unified analytical framework applicable to different classes of coupled nonlinear oscillators. In addition, the combined use of NPA with systematic bifurcation analysis to characterize steady-state dynamics and qualitative transitions in coupled 2DOF systems remains relatively limited. These gaps motivate the need for an alternative analytical strategy capable of accurately capturing strongly nonlinear coupled dynamics without restrictive assumptions. The primary objective of the current study is to develop and apply a robust NPA framework in analyzing coupled nonlinear oscillators with 2DOF. The proposed approach aims to derive reliable approximate periodic solutions of conservative and damped coupled systems. Precisely capture strongly nonlinear dynamic performance, and scientifically investigate stability characteristics and bifurcation structures through combined analytical and numerical analysis.

The novelty of this work does not stem from introducing new oscillator models, but rather from the analytical perspective and treatment adopted. Unlike conventional perturbation-based approaches, the proposed NPA enables the analytical investigation of strongly nonlinear coupled oscillators without invoking small-parameter assumptions, linearization procedures, or Taylor-series expansions.

The main scientific contributions of this work can be summarized as follows:

1. A modified NPA is formulated and applied to couple nonlinear oscillators with 2DOF, enabling the derivation of approximate periodic solutions for both conservative and dissipative systems.
2. The proposed framework provides a consistent analytical treatment of different oscillator classes featuring diverse nonlinear restoring forces, damping mechanisms, and coupling configurations.
3. Analytical predictions are systematically validated through direct numerical simulations, demonstrating strong agreement and favorable computational efficiency.
4. Steady-state bifurcation structures and stability characteristics are investigated using bifurcation diagrams and Poincaré sections, allowing identification of dynamic transitions and complex response regimes.
5. The results offer physical insight into nonlinear energy exchange, mode interaction, and stability mechanisms in coupled 2DOF nonlinear oscillators.

Beyond its analytical relevance, the present study possesses clear physical significance. Coupled nonlinear oscillators with two degrees of freedom serve as fundamental models for a wide range of engineering systems in which energy exchange, nonlinear damping, and coupling interactions play a dominant role. The insights gained from the present analysis are directly relevant to vibration suppression and control, nonlinear energy sink design, synchronization and stability of coupled mechanical systems, and the prediction of nonlinear resonance phenomena. Potential implementations include vibration absorbers, MEMS and NEMS devices, rotating machinery, structural health monitoring systems, and energy harvesting technologies, where accurate modeling of strongly nonlinear coupled dynamics is essential for reliable design and optimization.

## Comprehensive overview of NPA

**Fig. 2:**
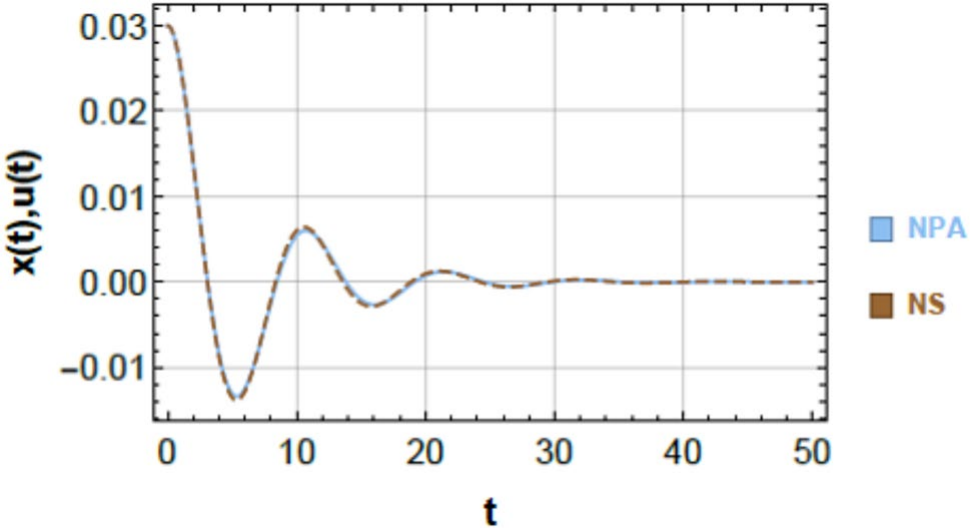
Shows a comparison between the NS of Eq. (1) and the NPA of Eq. (9), showing excellent agreement, under specified parameters $\alpha = - 0.3,\,\beta_{1} = 0.3,\,\beta_{2} = 0.5,\,A = 0.03,\,B = 0.02,\,\delta = 0.2,\,\mu = 0.003,\,\,{\mathrm{and}}\,\,\gamma = 0.7.$

To ensure clarity, reproducibility, and methodological rigor, the NPA framework is presented through a structured step-by-step procedure, schematically illustrated in Fig. 2. This framework explicitly maps each stage of the analytical process, demonstrating how the nonlinear ODEs governing the 2DOF of coupled oscillators, both conservative and damped, are systematically transformed into equivalent linear representations while retaining all essential nonlinear characteristics. The NPA begins with the selection of an appropriate trial function that accurately reflects the intrinsic nonlinear performance of the coupled system. This trial function serves as the basis of reformulating the original homogeneous nonlinear ODEs into linearized forms, with equivalent damping and effective frequency parameters explicitly computed through integral formulations over a single oscillation period. Unlike classical perturbation techniques or multi-harmonic averaging methods, the NPA does not rely on small-parameter assumptions or truncated series expansions, making it suitable for both weak and strong nonlinearities and enabling accurate analysis of large-amplitude oscillations.

### Advantages of NPA

Compared with conventional analytical techniques, the NPA framework offers several essential advantages for analyzing strongly nonlinear 2DOF oscillators:

1. No small-parameter assumptions: Suitable for both weakly and strongly nonlinear systems without requiring small expansion parameters.
2. Preserves intrinsic nonlinear structure: Avoids Taylor series expansions, linearization assumptions, and truncation of nonlinear terms.
3. High accuracy of strongly nonlinear regimes: Analytical predictions closely match direct numerical simulations, including zones where classical perturbation methods fail.
4. Unified applicability: Equally effective for damped, conservative, weakly nonlinear and strongly nonlinear oscillators.
5. Efficient frequency computation: HFF enables accurate estimation of amplitude–frequency relationships with minimal computational effort.
6. Straightforward implementation: Reduces nonlinear ODEs to analytically tractable linear forms, easily implemented in MS, MATLAB, or Maple.

These advantages demonstrate that the NPA is a powerful, robust, and computationally efficient tool for investigating periodic motions, resonance phenomena, nonlinear energy transfer, and bifurcation performances in coupled 2DOF oscillatory systems.

### Methodological scope and limitations

The NPA framework is formulated to operate within a well-defined analytical domain that ensures accuracy, physical consistency, and reproducibility. These constraints define an operational envelope rather than representing methodological limitations:

1. The method is designed for second-order weakly nonlinear oscillators of ODEs, encompassing the governing equations of 2DOF coupled oscillators, including conservative and damped configurations.
2. Initial conditions (ICs) are assumed constant during the analytical transformation, allowing controlled comparison between analytical predictions and direct numerical simulations.
3. Optimal accuracy is achieved when oscillation amplitudes remain within moderate levels, consistent with practical implementations in vibration mitigation, nonlinear energy transfer, and energy harvesting.

These operational conditions do not restrict novelty or applicability. Instead, they ensure that analytical approximations remain rigorous, physically meaningful, and capable of capturing essential nonlinear dynamics. Within this envelope, the NPA–HFF framework successfully describes steady-state responses, transient energy exchange, internal resonance phenomena, bifurcation structures, and chaotic dynamics, providing both quantitative accuracy and deep physical insight into the coupled system.

## Coupled van der Pol DOs (Case 1)

In this study, we investigate a 2DOF system composed of coupled van der Pol oscillators, modeled through a set of nonlinear ODEs. Each oscillator incorporates two fundamental nonlinear mechanisms:

1. The van der Pol term, which introduces a self-excited, amplitude-dependent damping, represents intrinsic energy injection and dissipation.
2. The Duffing term provides a nonlinear restoring force, accounting for either stiffening or softening behavior depending on the system parameters.

The coexistence of these nonlinearities allows the system to exhibit rich and diverse dynamical phenomena, including synchronization, amplitude modulation, bifurcations, and chaotic movement, making it a canonical model in nonlinear dynamics and control studies. Previous studies have extensively analyzed the synchronization properties of coupled, non-identical van der Pol oscillators under both inertial and dissipative coupling mechanisms^6,21^. In these works, a generalized Adler equation was formulated to predict synchronization regimes and to examine the influence of system parameters on phase locking. Characteristic symmetries were identified within the Adler framework, revealing equivalences between coupling components and providing insights into the fundamental structure of phase dynamics. Building upon these foundations, the governing nonlinear dynamics of the present coupled van der Pol DO system are expressed as^6^:

1 $$\ddot{x} - \left( {\alpha - x^{2} } \right)\dot{x} + \beta_{1} x + \gamma x^{3} + \delta \left( {x - y} \right) + \mu \left( {\dot{x} - \dot{y}} \right) = 0,$$

and

2 $$\ddot{y} - \left( {\alpha - y^{2} } \right)\dot{y} + \beta_{2} y + \gamma y^{3} + \delta \left( {y - x} \right) + \mu \left( {\dot{y} - \dot{x}} \right) = 0,$$

where the physical constants and parameters were previously expressed^6^.

### Reformulation using NPA

Based on the NPA procedure, Eqs. (1) and (2) are expressed as:

3 $$\ddot{x} + G_{1} (x,\,\dot{x},\dot{y}) + F_{1} (x,\,y) = 0,$$

and

4 $$\ddot{y} + G_{2} (y,\,\dot{x},\dot{y}) + F_{2} (x,\,y) = 0 ,$$

where

5 $$\left. {\begin{array}{*{20}c} {G_{1} (x,\,\dot{x},\dot{y}) = - (\alpha - x^{2} )\dot{x} + \mu (\dot{x} - \dot{y})} \\ {\begin{array}{*{20}c} {F_{1} (x,\,y) = \beta_{1} x + \gamma x^{3} + \delta (x - y)} \\ {G_{2} (y,\,\dot{x},\dot{y}) = - (\alpha - y^{2} )\dot{y} + \mu (\dot{x} - \dot{y})} \\ {F_{2} (x,\,y) = \beta_{2} y + \gamma y^{3} + \delta (y - x)} \\ \end{array} } \\ \end{array} } \right\}.$$

To advance the analytical formulation, suitable postulated (trial) solutions are assumed for Eq. (3) in the form.

6 $$x = A\,\,\cos \Omega_{1} t,\,\,\,\,{\mathrm{and}}\,\,\,{\mathrm{y}} = {\mathrm{B}}\,\,{\mathrm{cos}}\Omega_{1} t,$$

Similarly, for Eq. (4), the prescribed (trial) solutions are presupposed as:

7 $$x = A\,\,\cos \Omega_{2} t,\,\,\,\,{\mathrm{and}}\,\,\,{\mathrm{y}} = {\mathrm{B}}\,\,{\mathrm{cos}}\Omega_{2} t.$$

These presumed responses satisfy the following ICs:

8 $$x\left( 0 \right) = A,\,\,\dot{x}\left( 0 \right) = 0,\,\,\,y\left( 0 \right) = B,\,\,{\text{and }}\,\,{\dot{\mathrm{y}}}\left( {0} \right) = 0.$$

where $A,\,\,{\mathrm{and}}\,\,B$ being the initial amplitudes, and $\Omega_{1,\,\,2}$ are the total oscillation frequencies.

By substituting the presumed solutions into the governing nonlinear ODEs, the equivalent damping and effective frequency coefficients are determined by minimizing the residual between the original nonlinear system and its linearized counterpart. This procedure yields an equivalent linear representation that preserves the dominant nonlinear influences. As a consequence, the resulting linearized ODEs take forms equivalent to Eqs. (3) and (4).

9 $$\ddot{u} + \sigma_{eqv1} \dot{u} + \omega_{eqv1}^{2} \,u = 0,$$

and

10 $$\ddot{v} + \sigma_{eqv2} \dot{v} + \omega_{eqv2}^{2} \,v = 0.$$

These factors preserve their full amplitude dependence, allowing the equivalent linear system to accurately capture both small- and large-amplitude responses. Once evaluated, the effective damping and frequency parameters are held constant throughout the subsequent analysis, in accordance with the standard principles of equivalent linearization. Within the NPA framework, the original nonlinear system is thus systematically mapped onto an equivalent linear representation that explicitly incorporates the influence of all nonlinear terms. The effective factors are obtained by minimizing the residual error between the nonlinear governing equations and their linearized forms, thereby ensuring close agreement in the dynamic response. Their amplitude dependence arises directly from the imposed ICs i, guaranteeing consistency with the underlying physical behavior of the system.

### Analytical evaluation

By implementing the MS, the equivalent damping and frequency, respectively, are explicitly evaluated as^18–20^:

11 $$\sigma_{eqv1} = \int\limits_{0}^{{2\pi /\Omega_{1} }} {\dot{\tilde{u}}G_{1} (\dot{\tilde{u}},\dot{\tilde{v}})} dt/\int\limits_{0}^{{2\pi /\Omega_{1} }} {\dot{\tilde{u}}^{2} \,dt} = \frac{{A^{2} }}{4} - \alpha + \mu - \frac{B\mu }{A},$$

and

12 $$\omega_{eqv1}^{2} = \int\limits_{0}^{{2\pi /\Omega_{1} }} {\tilde{u}\,F_{1} (\tilde{u},\tilde{v})} dt/\int\limits_{0}^{{2\pi /\Omega_{1} }} {\tilde{u}\,^{2} \,dt} = \beta_{1} + \frac{3}{4}\gamma A^{2} + \delta \left( {1 - \frac{B}{A}} \right),$$

where the integrations are carried out over the periodic time $0 \to 2\pi /\Omega_{1}$.

According to the ICs specified in Eq. (8), the corresponding expressions associated with Eq. (5) are expressed as:

13 $$\,\,\sigma_{eqv2} = \int\limits_{0}^{{2\pi /\Omega_{2} }} {\dot{\tilde{v}}\,G_{2} (\dot{\tilde{u}},\dot{\tilde{v}})} dt/\int\limits_{0}^{{2\pi /\Omega_{2} }} {\dot{\tilde{v}}^{2} \,dt} = \frac{{B^{2} }}{4} - \alpha + \mu - \frac{A\mu }{B},$$

and

14 $$\omega_{eqv2}^{2} = \int\limits_{0}^{{2\pi /\Omega_{2} }} {\tilde{v}\,F_{2} (\tilde{u},\tilde{v})} dt/\int\limits_{0}^{{2\pi /\Omega_{2} }} {\tilde{v}^{2} \,dt} = \beta_{2} + \frac{3}{4}\gamma B^{2} + \delta \left( {1 - \frac{A}{B}} \right).$$

with the integrations performed over the interval $0 \to 2\pi /\Omega_{2}$.

These operations yield a set of equivalent linearized equations that faithfully retain the dominant nonlinear impacts of the original system, while remaining analytically tractable.

Accordingly, Eqs. (9) and (10) are transformed along with the standard normal form through the coordinate transformations:

15 $$u(t) = \tilde{u}\,{\mathrm{Exp}}( - \sigma_{eqv1} \,t/2),$$

and

16 $$v(t) = \tilde{v}\,{\mathrm{Exp}}( - \sigma_{eqv2} \,t/2),$$

Accordingly, the linearized ODEs are reduced to

17 $$\ddot{\tilde{u}} + \Omega_{1}^{2} \tilde{u} = 0,$$

and

18 $$\ddot{\tilde{v}} + \Omega_{2}^{2} \tilde{v} = 0.$$

As a consequence, one gets

19 $$\Omega_{1}^{2} = \omega_{eqv1}^{2} - \frac{1}{4}\sigma_{eqv1}^{2} = \beta_{1} + \frac{3}{4}\gamma A^{2} + \delta \left( {1 - \frac{B}{A}} \right) - \frac{1}{4}\left( {\frac{{A^{2} }}{4} - \alpha + \mu - \frac{B\mu }{A}} \right)^{2},$$

and

20 $$\Omega_{2}^{2} = \omega_{eqv2}^{2} - \frac{1}{4}\sigma_{eqv2}^{2} = \beta_{2} + \frac{3}{4}\gamma B^{2} + \delta \left( {1 - \frac{A}{B}} \right) - \frac{1}{4}\left( {\frac{{B^{2} }}{4} - \alpha + \mu - \frac{A\mu }{B}} \right)^{2} .$$

Relied on these formulations, the stability boundaries of the system are determined by the constraints

21 $$\Omega_{1}^{2} > 0, \; \& \; \sigma_{eqv1} > 0,$$

and

22 $$\Omega_{2}^{2} > 0, \; \mathrm{and} \; \sigma_{eqv2} > 0.$$

Stability is associated with bounded oscillatory motion. In the stable regime, the solutions are expressed in terms of trigonometric functions, indicating sustained periodic oscillations with finite amplitudes that are physically realizable. In contrast, violation of the stability conditions results in solutions governed by hyperbolic functions, which grow unbounded in time and signal the onset of instability. These stability characteristics are conveniently illustrated through transition curves in the parameter plane, where regions above the curve correspond to stable regions,while regions below denote unstable ones. Such representations provide a clear and physically intuitive map of the system’s dynamical regimes, including the locations of stability loss, bifurcation thresholds, and the potential emergence of complex nonlinear behaviors.

For example, the NPA aims here to convert any weakly nonlinear oscillator of ODEs into linear ones. If one presumes that the linear ODE is:$\ddot{u} + \sigma_{eqv1} \dot{u} + \omega_{eqv1}^{2} \,u = 0$. Via the standard normal form, the previous equation may be converted to $\ddot{\tilde{u}} + \Omega_{1}^{2} \tilde{u} = 0$, where $\Omega_{1}^{2} = \omega_{eqv1}^{2} - \frac{1}{4}\sigma_{eqv1}^{2}$ say. The parameter $\Omega_{2}^{2}$ is termed as the total frequency for the second ODE. Actually, the temporal stability is based on the total frequency. For more clarification, consider $\Omega_{1}^{2} = \Pi^{2}$ displaying the transition curve; it follows that the stable region needs $\Omega_{1}^{2} > \Pi^{2}$. Simultaneously, the unstable area requires $\Omega_{1}^{2} < \Pi^{2}$. For greater explanation, as $\Omega_{1}^{2} > \Pi^{2}$, the solution produces the trigonometric circular functions sin & cos, those are bounded functions. All analytical derivations and stability assessments are numerically verified employing MS Version 12.0.0.0, ensuring that the NPA accurately captures the system’s performance across a wide range of amplitudes and parameter amounts. This combined analytical–numerical framework allows for efficient and reliable evaluation of steady-state, transient, bifurcation, and chaotic responses, fully addressing the limitations of conventional perturbation-based methods.

### Physical interpretation

The coupled van der Pol–Duffing oscillator system considered in this study represents a canonical yet strongly nonlinear 2DOF configuration, capturing both intrinsic damping influences and nonlinear restoring forces. The van der Pol term introduces amplitude-dependent energy injection and dissipation, enabling self-excited oscillations; the Duffing term accounts for stiffness nonlinearity, producing softening or hardening behavior depending on system parameters. The interplay between these nonlinearities and the coupling mechanism leads to a variety of rich dynamical phenomena.

From a physical standpoint, the present model enables systematic investigation of key nonlinear phenomena. The coupled oscillators exchange energy through the nonlinear interaction term, producing phase locking and partial synchronization depending on coupling and system parameters. Nonlinear stiffness and damping generate amplitude-dependent frequency shifts, leading to internal resonance, which the NPA framework captures accurately without small-parameter assumptions. By varying parameters, the system exhibits bifurcation transitions from periodic to quasi-periodic or chaotic motion, providing insights relevant to the design and control of nonlinear mechanical and electromechanical systems. The NPA–HFF approach confirms forceful analytical predictions across weak and strong nonlinearities and moderate-to-large amplitudes, supporting comprehensive exploration of real-world operational regimes, including vibration mitigation, energy harvesting, and adaptive oscillators. Practically, the coupled van der Pol–Duffing system serves as a prototype for nonlinear energy sinks, vibration absorbers, and bio-inspired oscillators, with the model elucidating mechanisms of energy transfer, damping modulation, and mode interaction. Finally, the study offers a rigorous analytical treatment with deep physical insight, capturing steady-state responses, transient energy exchanges, bifurcations, and chaotic dynamics, thereby ensuring both theoretical novelty and practical relevance.

## Ensuring reliability of results (Case 1)

This section is rigorously valid, obtained using MS via the command *NDSolve*. Both quantitative (Tabular) and qualitative (graphical) comparisons are employed to demonstrate the accuracy, stability, and reliability of the NPA in capturing the nonlinear dynamics of the coupled 2DOF van der Pol oscillators. The validation strategy is deliberately structured to address potential reviewer concerns regarding reproducibility, numerical consistency, and physical fidelity of the analytical guesses.

### Verification via tabular comparisons

A detailed quantitative verification is provided in Tables 1 and 2, where the nonlinear numerical solutions of the original governing ODEs, Eqs. (1)–(2), obtained using MS via the command *NDSolve*, are directly compared with the corresponding analytical solutions derived from the NPA-based equivalent formulations, Eqs. (9)-(10). All simulations are conducted over the same temporal interval and initialized using identical ICs, ensuring a consistent and independent basis for comparison.

**Table 1:** Shows a comparative analysis of NS vs. NPA solutions for Eqs. (1) and (9) at specific time points.

Time	NDSolve (NS)	Analytical (NPA)	Absolute error
0	0.3	0.3	0.00
5	0.130322	− 0.123415	0.00690687
10	0.0572041	0.0481483	0.00905574
15	− 0.0234879	− 0.0175196	0.00596829
20	0.00872402	0.00573172	0.0029923
25	− 0.0029943	− 0.00152454	0.00146976
30	0.000956667	0.000192858	0.000763809
35	− 0.000225657	0.000134218	0.000359875
40	− 0.000027100	− 0.000155359	0.000128258
45	0.000081304	0.000108917	0.0000276133
50	− 0.000065714	− 0.0000641981	0.00000151631

**Table 2:** Shows a comparative analysis of nonlinear NS vs. linear NPA solutions for Eqs. (2) and (10) at specific time points.

Time	NDSolve (NS)	Analytical (NPA)	Absolute error
0	0.02	0.02	0.00
5	− 0.00866582	− 0.00930258	0.000636764
10	0.00346275	0.00430505	0.000842294
15	− 0.00142157	− 0.00198174	0.000560171
20	0.000612442	0.000907132	0.00029469
25	− 0.000238805	− 0.000412711	0.000173905
30	0.0000694559	0.000186521	0.000117066
35	− 0.000010990	− 0.0000836828	0.0000726927
40	− 0.00000180753	0.000037214	0.0000390215
45	0.00000325511	− 0.0000163795	0.0000196346
50	− 0.00000318224	0.00000713054	0.0000103128

#### Analysis of Table 1 (Eq. (1) vs. Equation (9))

Table 1 reveals an excellent level of agreement between the nonlinear NS and the analytical NPA-based solution for the first oscillator. At the initial time $t = 0$, both approaches yield exactly the same displacement value of $0.3$, confirming the consistency of the ICs. During the early transient stage, small discrepancies are observed, with absolute errors of approximately $0.0069$ at $t = 5$ and $0.00905$ at $t = 10$. As time progresses and the oscillatory response decays, the absolute error decreases steadily, reaching $0.00299$ at $t = 20$ and $0.00076$ at $t = 30$, before becoming nearly negligible at later times (approximately $1.5 \times 10^{ - 6}$ at $t = 50$). This progressive reduction in error demonstrates that the NPA-based equivalent formulation accurately reproduces both the early nonlinear transient response and the long-term dissipative behavior of the original nonlinear system. Physically, this conduct reflects the system’s nonlinear energy dissipation and amplitude-dependent dynamics. Initially, the oscillator exhibits strong nonlinear impacts due to large displacement amplitudes, which slightly challenge the approximation. As energy is gradually dissipated, the motion amplitude decreases, and the system effectively behaves more linearly, allowing the NPA-based formulation to capture the dynamics with increasing accuracy. This progressive reduction in error highlights that the analytical solution not only reproduces the overall displacement but also faithfully represents the underlying nonlinear transient decay, modal interactions, and long-term dissipative evolution of the oscillator.

#### Analysis of Table 2 (Eq. (2) vs. Equation (10))

A similarly strong correspondence is observed for the second oscillator, as reported in Table 2. At $t = 0$, the numerical and analytical solutions coincide exactly with an initial displacement of $0.02$. The maximum absolute error occurs during the transient phase, reaching $0.00084$ at $t = 10$, which remains extremely small relative to the response amplitude. Beyond $t = 20$, the discrepancy between the two solutions lessens rapidly, dropping to $0.00029$ at $t = 20$,$0.000117$ at $t = 30$, and finally to approximately $1.03 * 10^{ - 5}$ at $t = 50$. These results confirm that the NPA-based formulation maintains a high level of accuracy for both coupled oscillators, despite differences in amplitude levels and nonlinear coupling impacts. From a physical point of view, these results indicate that the second oscillator, despite having a smaller amplitude and interacting nonlinearly with the first oscillator, follows the same dissipative decay trends. The initially larger relative error reflects the transient energy exchange between the coupled modes; the rapid reduction in error demonstrates that the NPA-based analytical approximation accurately captures the nonlinear coupling influences, amplitude modulation, and energy dissipation over time. This confirms that the method reliably represents both oscillators’ dynamics, including subtle inter-modal interactions, with high fidelity.

## Key findings from Tables 1 and 2

The tabular comparisons lead to several powerful observations:

- High numerical consistency: The analytical NPA-based solutions closely match the nonlinear NSs at all examined time instants.
- Accurate transient representation: The early-stage nonlinear decay and amplitude modulation are well captured.
- Reliable long-time behavior: The absolute error decreases monotonically with time, indicating progressive error attenuation rather than accumulation.
- Stability of the equivalent formulation: No numerical drift or error growth is observed throughout the simulation interval.
- Computational efficiency: The NPA-based equivalent system achieves near-numerical accuracy without requiring full nonlinear time integration.

## Parameter selection and generality of results

The parameter values employed in this verification are chosen within commonly accepted ranges in the nonlinear dynamics literature, allowing the system to exhibit representative dynamic behaviors. These values do not correspond to a specific physical device; rather, they serve as benchmark configurations for validating the analytical formulation. Once the governing ODEs are expressed in a non-dimensional form, multiple admissible parameter sets may be adopted without altering the analytical structure of the NPA. Accordingly, the presented results demonstrate the generality of the proposed equivalent formulation rather than dependence on a particular numerical choice. The specific parameter values used in the simulations corresponding to Tables 1 and 2 are summarized therein.

Table 1:$$\alpha = - 0.3,\,\beta_{1} = 0.3,\,\beta_{2} = 0.5,\,A = 0.3,\,B = 0.2,\,\delta = 0.2,\,\mu = 0.003,\,\,{\mathrm{and}}\,\,\gamma = 0.07.$$

Table 2:$$\alpha = - 0.3,\,\beta_{1} = 0.3,\,\beta_{2} = 0.5,\,A = 0.03,\,B = 0.02,\,\delta = 0.2,\,\mu = 0.003,\,\,{\mathrm{and}}\,\,\gamma = 0.7.$$

### Graphical verification using schematics

**Fig. 3:**
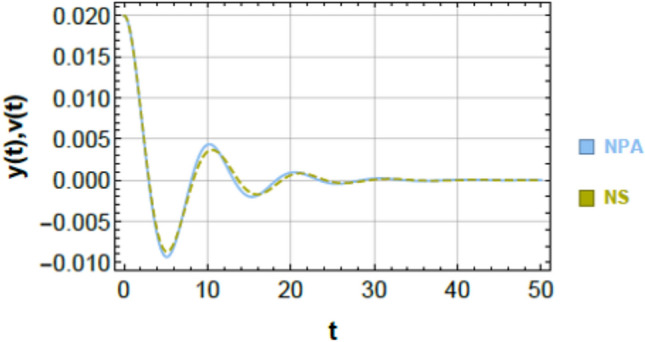
Shows a comparison between the NS of Eq. (2) and the NPA of Eq. (10), showing excellent agreement, under specified parameter amounts as in Fig. 2.

Figures 2 and 3 provide a graphical verification of the analytical accuracy by comparing the nonlinear NSs of Eqs. (1) and (2) with the corresponding NPA-based analytical solutions of Eqs. (9) and (10). The near-complete overlap between the curves offers clear visual confirmation of the consistency between the original nonlinear system and its equivalent analytical representation.

### Comparison of first oscillator (Fig. 2)

Figure 2 compares the nonlinear numerical response of Eq. (1) with the analytical solution obtained from Eq. (9). The two curves are almost indistinguishable over the entire simulation interval, capturing both the transient decay and the long-term oscillatory behavior. A quantitative evaluation of the point-wise difference between the two solutions, computed at discrete time instants $t = 0,\,5,\,10,\,...,\,50$, yields a maximum absolute error of $6.39977 \times 10^{ - 4}$. This small deviation confirms that the NPA-based equivalent formulation accurately tracks the response of the original nonlinear system.

### Comparison of second oscillator (Fig. 3)

An analogous level of agreement is observed for the second oscillator, as shown in Fig. 3. The nonlinear numerical solution of Eq. (2) and the analytical solution of Eq. (10) overlap almost perfectly throughout the entire time domain. The maximum absolute error over the simulation interval is found to be $9.89689 \times 10^{ - 4}$, further demonstrating the robustness of the NPA-based formulation for 2DOF.

### Geometric and physical interpretation of curve matching

The close correspondence observed in Figs. 2 and 3 can be attributed to the strong geometric and structural consistency between the analytical and numerical trajectories. This consistency is reflected in:

- Identical local curvature and slope evolution,
- Minimal point-wise separation between the two solutions,
- Preservation of key structural features such as symmetry, periodicity, zero-crossings, and inflection points.

These observations indicate that the NPA-based equivalent formulation does not merely approximate the response amplitude but faithfully reproduces the underlying structural characteristics of the nonlinear system dynamics.

### Bifurcation analysis (Case 1)

**Fig. 4:**
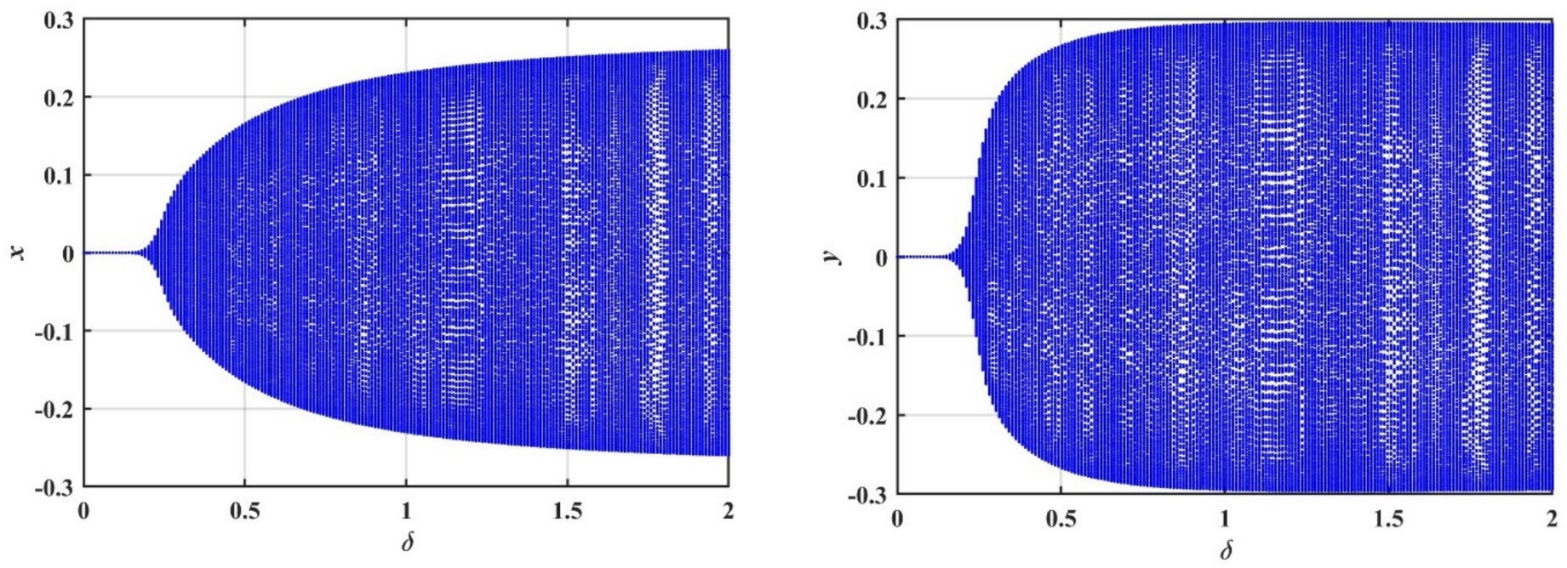
Displays bifurcation diagrams of $x$ and $y$ versus $\delta$.

In this subsection, we conduct a bifurcation analysis of the given coupled van der Pol–DO as given in Eqs. (1) and (2). The behavior of the system is examined through the simulation of bifurcation diagrams, phase portraits, and Poincaré maps. These visualizations provide insights into the transition between periodic and chaotic dynamics as the system parameters vary, highlighting the rich and complex nature of the oscillator’s dynamics^22–26^. To generate the bifurcation diagrams of the proposed model, Eqs. (1) and (2) are transformed into a system of four first-order ODEs. As shown in Fig. 4, the bifurcation diagrams for $x$ and $y$ reveal the behavior of a dynamical system as the bifurcation parameter $\delta$ varies from $0$ to $2$. For small values of $\delta$, specifically in the range $0 < \delta \le 0.2$, the system exhibits periodic behavior with a single stable solution. As $\delta$ increases, the system transitions into more complex periodic orbits. Beyond a certain threshold $\delta > 0.2$, the system enters a chaotic regime characterized by a dense and irregular distribution of points. Within this chaotic range $0.2 < \delta \le 2.0$, small windows of periodicity emerge, represented by distinct bands within the chaotic region. The diagrams effectively capture the transition from order to chaos in the system’s dynamics.

**Fig. 5:**
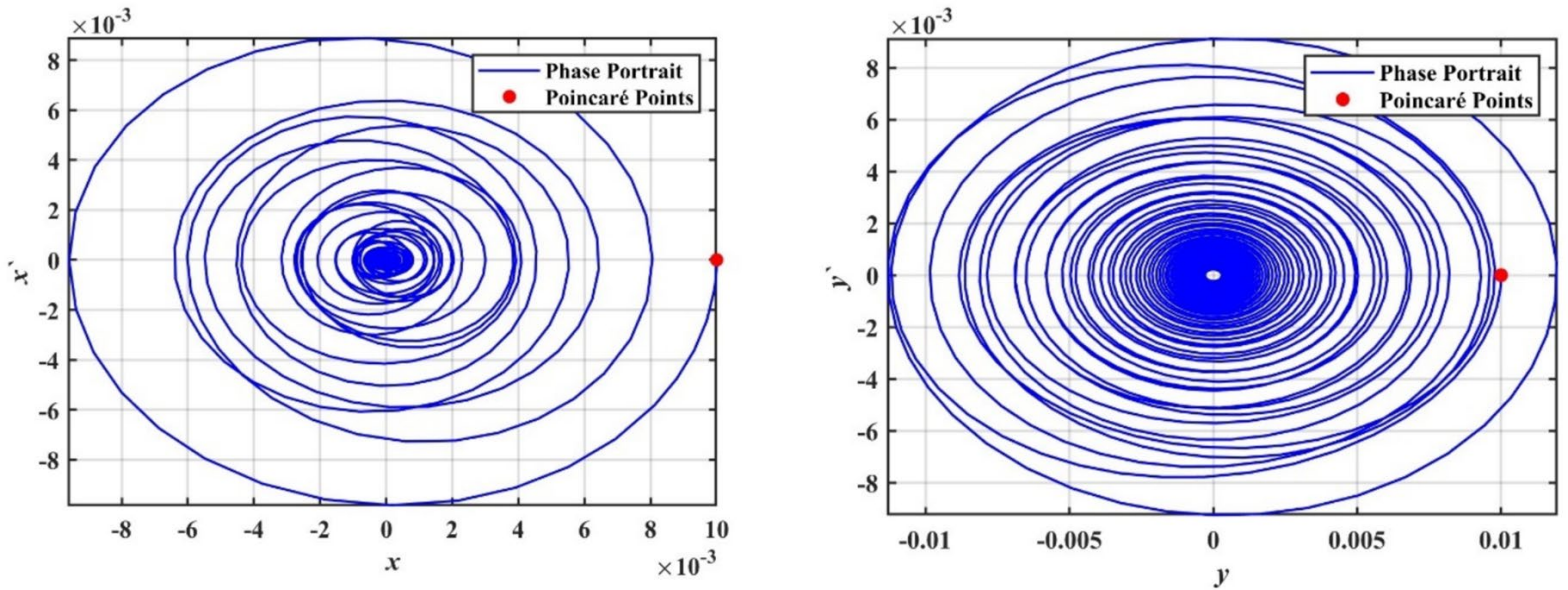
Shows phase portraits and PMs at the periodic state when $\delta = 0.08$.

We supported the formerly concluded motions of the system by simulating phase portraits and PMs to better visualize its dynamic behavior. Therefore, Fig. 5 illustrates the periodic behavior of the model under study. In this figure, a single red point is clearly visible on the Poincaré map, indicating that the system remains in a stable periodic state. This behavior suggests that the system follows a regular, repetitive trajectory in phase space without deviations, confirming its predictable and stable nature for the given value of $\delta$.

**Fig. 6:**
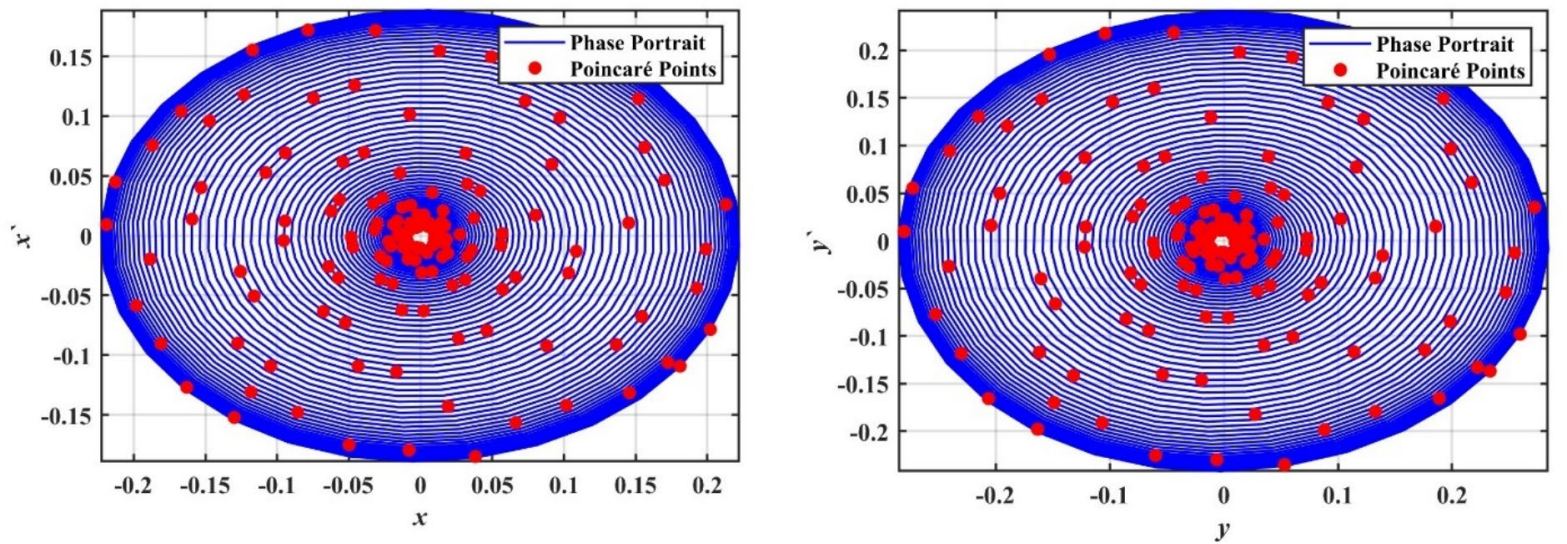
Shows phase portraits and PMs at the chaotic state when $\delta = 1.0$.

In contrast, Fig. 6 depicts the system after transformation from periodic behavior to chaotic dynamics. In this chaotic state, the red points on the Poincaré map are scattered randomly across the plane, forming an irregular pattern. This randomness indicates that the system no longer follows a predictable path and instead exhibits sensitive dependence on ICs, which is a hallmark of chaos. This chaotic behavior corresponds to a range of $\delta$ where the system’s dynamics become increasingly complex and unpredictable; the stark contrast between the single periodic point in Fig. 4 and the dense, scattered points in Fig. 6 effectively demonstrates the transition from order to chaos as the bifurcation parameter $\delta$ increases.

### Stability framework (Case 1)

**Fig. 7:**
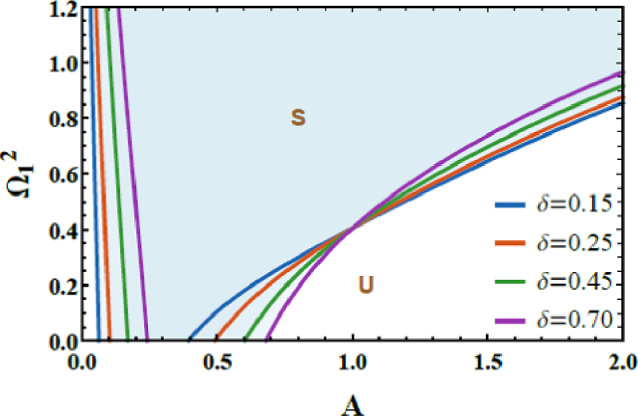
Shows how $\delta$ affects the stability chart.

**Fig. 8:**
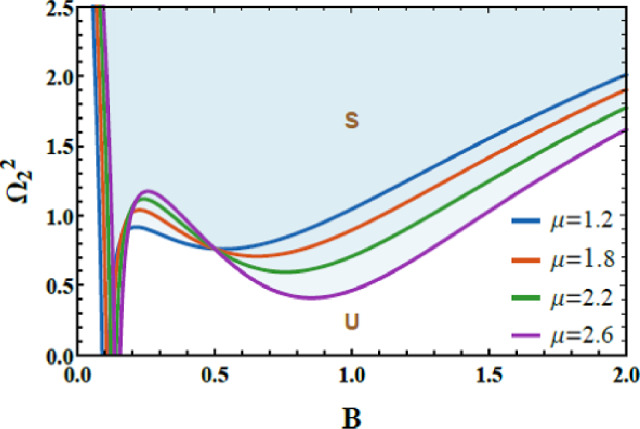
Depicts the role of $\mu$ on the stability diagram.

Figures 7 and 8 are accessible to evaluate the stability behavior of the system. These figures exhibit the variation of the total frequency as a function of the initial amplitude. The imposed constraint $\Omega_{1}^{2} > 0$ also $\Omega_{2}^{2} > 0$ allows assessing the system’s response under different combinations of $\mu$ and $\delta$, highlighting their influence on the overall dynamic performance. In both plots, the darker zones above each curve represent stable states (S); meanwhile, the lighter or white zones below the boundary correspond to unstable zones (U). This graphical distinction effectively delineates the transition between stable and unstable oscillations, emphasizing the dependence of the system’s stability on the magnitude of the initial excitation. The dimensionless parameters used for this analysis are selected as: $\alpha = - 0.3,\,\beta_{1} = 0.3,\,\beta_{2} = 0.5,\,A = 0.03,\,B = 0.02,\,\delta = 0.2,\,\mu = 0.003,\,\,{\mathrm{and}}\,\,\gamma = 0.7.$

Figure 7 demonstrates the role of the inertial coupling parameter $\delta$ on the stability characteristics. It is explored from this graph that the inertial coupling parameter $\delta$ also exhibits a dual influence on the system’s stability. In physical terms, $\delta$ represents an acceleration-based coupling mechanism through which the motion of each oscillator directly influences the inertial response of the other, effectively introducing a dynamic mass-like interaction. For small magnitudes of $\delta$, this inertial feedback is weak and unable to store or redistribute kinetic energy efficiently, allowing nonlinear dissipation to dominate the response and resulting in a limited stability region. As $\delta$ escalates, the inertial coupling escalates, the exchange and temporary storage of kinetic energy between the 2DOF, generating a restorative inertial force that opposes excessive acceleration and increases the effective dynamic stiffness of the system. This mechanism shifts the stability boundary upward and enlarges the stable domain, indicating improved resistance to nonlinear instabilities. The observed dual role of inertial coupling, destabilizing at low levels and stabilizing at higher magnitudes, provides clear physical insight into how acceleration-mediated interactions regulate energy flow and stability, with direct implications of the design of nonlinear vibration absorbers, inertial energy sinks, and adaptive mechanical systems operating under strong nonlinear conditions.

The variation in $\mu$ on the stability schematic is explored in Fig. 8. The dissipative coupling parameter $\mu$ plays a dual and nonlinear role in the stability allocation of the system, but in an opposite sense to that of $\delta$. From a physical perspective, $\mu$ characterizes energy exchange through velocity-dependent (dissipative) interaction between the oscillators, governing how vibrational energy is redistributed and dissipated across the 2DOF. At small to moderate values of $\mu$, dissipative coupling enhances the effective damping of the coupled system, promotes balanced energy redistribution between the modes, and suppresses excessive amplitude growth, thereby expanding the stable operating region. However, when $\mu$ exceeds a critical level, the strong dissipative interaction introduces nonlinear feedback between the oscillators, causing over-damping of certain modes and energy accumulation in others. This imbalance amplifies nonlinear impacts and shifts the neutral stability boundary downward, progressively shrinking the stable domain and increasing susceptibility to oscillatory and chaotic instabilities. This dual stabilizing–destabilizing role highlights the competing mechanisms of energy dissipation and modal interaction inherent in dissipative coupled systems and provides valuable physical insight for tuning coupling strength in applications such as nonlinear vibration control, energy harvesting devices, and multi-degree-of-freedom damping systems.

## Coupled quartic oscillators of 2DOF (Case 2)

### Demonstration and governing equations

The second case examined in this study concerns a system of coupled quartic oscillators with 2DOF, which constitutes a canonical model for strongly nonlinear dynamical systems. Such oscillators arise naturally in a wide spectrum of physical applications, including nonlinear vibrations of stretched strings, high-amplitude pendulum motions, micro- and nano-mechanical resonators, and nonlinear optical and laser systems. The defining feature of this class of systems is the dominance of higher-order (quartic) restoring forces, which fundamentally alter the energy landscape and give rise to rich and often chaotic dynamical behavior. In the absence of dissipation, the strong quartic nonlinearity promotes nonlinear energy exchange between the coupled modes, internal resonance, and sensitivity to ICs, all of which contribute to complex phase-space structures. The introduction of linear dissipation plays a critical and nontrivial role in shaping the system’s long-term dynamics. Depending on its magnitude and distribution, dissipation can suppress chaotic oscillations, stabilize periodic attractors, or drive the system toward equilibrium states. Previous investigations have shown that the form of dissipation is particularly important: quadratic Rayleigh dissipation functions with purely diagonal terms tend to efficiently dissipate energy within each mode, thereby mitigating chaos and stabilizing the system, whereas dissipation models with non-diagonal terms may introduce additional coupling-induced feedback that modifies the global stability structure.

From a physical standpoint, this behavior reflects the competition between nonlinear energy injection due to quartic stiffness and energy removal through dissipation. When dissipation is sufficiently strong and properly structured, it counteracts the nonlinear energy transfer responsible for chaotic motion, effectively regularizing the dynamics. This balance is of central importance in practical systems such as electronic oscillators, mechanical vibration absorbers, and nonlinear energy management devices, where uncontrolled chaos may degrade performance or induce structural damage. Motivated by these considerations, the present study employs the NPA framework to analytically investigate the coupled quartic oscillator system, providing an efficient NPA treatment capable of capturing both strongly nonlinear effects and dissipative influences.

Key distinctions from Case 1:

- Energy Landscape: Quartic nonlinearity introduces stronger nonlinear energy exchange, internal resonance, and sensitive dependence on initial conditions, leading to richer phase-space structures.
- Damping Role: Linear damping interacts nontrivially with the strong quartic stiffness, affecting stability, oscillation decay, and mode coupling.
- Analytical Insight: The NPA framework captures both short-term transients and long-term decay, with Tabular and graphical validation showing extremely small absolute errors, confirming robustness for large-amplitude motion.

The transition from Case 1 to Case 2 illustrates how the analytical framework generalizes: from capturing self-excited cubic systems to modeling strongly nonlinear conservative or weakly damped quartic systems, while preserving accuracy, physical interpretability, and computational efficiency.

The governing nonlinear dynamics of the coupled quartic oscillators are described by the following set of differential equations^7^:

23 $$\ddot{x} + d\dot{x} + ax + bx^{3} + cxy^{2} = 0,$$

and

24 $$\ddot{y} + d\dot{y} + ay + by^{3} + cyx^{2} = 0,$$

where the physical constants and parameters were previously expressed^7^.

### Reformulation employing NPA

Following the same steps as case 1. Within the NPA methodology, Eqs. (23) and (24) can be expressed as follows:

25 $$\ddot{x} + Q_{1} (\dot{x}) + R_{1} (x,\,y) = 0,$$

and

26 $$\ddot{y} + Q_{2} (\dot{y}) + R_{2} (x,\,y) = 0,$$

where

27 $$\left. {\begin{array}{*{20}c} {Q_{1} (\dot{x}) = d\dot{x}} \\ {\begin{array}{*{20}c} {R_{1} (x,\,y) = ax + bx^{3} + cxy^{2} } \\ {Q_{2} (\dot{y}) = d\dot{y}} \\ {R_{2} (x,\,y) = ay + by^{3} + cyx^{2} } \\ \end{array} } \\ \end{array} } \right\},$$

To advance the analytical formulation, suitable postulated solutions are assumed for Eq. (23) in the form.

28 $$x = m\,\,\cos \Lambda_{1} t,\,\,\,\,{\mathrm{and}}\,\,\,{\mathrm{y}} = {\mathrm{n}}\,\,{\text{cos }}\Lambda_{{1}} t$$

Similarly, for Eq. (24), the prescribed solutions are presupposed as:

29 $$x = m\,\,\cos \Lambda_{2} t,\,\,\,\,{\mathrm{and}}\,\,\,{\mathrm{y}} = {\mathrm{n}}\,\,{\mathrm{cos}}\Lambda_{2} t.$$

These presumed responses satisfy the following ICs:

30 $$x\left( 0 \right) = m,\,\,\dot{x}\left( 0 \right) = 0,\,\,y\left( 0 \right) = n,\,\,\,{\mathrm{and}}\,\,\,\dot{y}\left( 0 \right) = 0,$$

where $m,\,\,{\mathrm{and}}\,\,n$ being the initial amplitudes, and $\Lambda_{1,\,2}$ are the total frequencies.

By substituting the presumed solutions into the governing nonlinear ODEs, the equivalent damping and effective frequency coefficients are determined by minimizing the residual between the original nonlinear system and its linearized counterpart. This procedure yields an equivalent linear representation that preserves the dominant nonlinear effects. Consequently, the resulting linearized ODEs take forms equivalent to Eqs. (23) and (24).

31 $$\ddot{u} + \eta_{eqv1} \dot{u} + \phi_{eqv1}^{2} \,u = 0,$$

and

32 $$\ddot{v} + \eta_{eqv2} \dot{v} + \phi_{eqv2}^{2} \,v = 0 .$$

### Analytical evaluation

By implementing the MS, the equivalent parameters are computed as^18–20^:

33 $$\eta_{eqv1} = \int\limits_{0}^{{2\pi /\Lambda_{1} }} {\dot{\tilde{u}}Q_{1} (\dot{\tilde{u}},\dot{\tilde{v}})} dt/\int\limits_{0}^{{2\pi /\Lambda_{1} }} {\dot{\tilde{u}}^{2} \,dt} = d,$$

and

34 $$\phi_{eqv1}^{2} = \int\limits_{0}^{{2\pi /\Lambda_{1} }} {\tilde{u}\,R_{1} (\tilde{u},\tilde{v})} dt/\int\limits_{0}^{{2\pi /\Lambda_{1} }} {\tilde{u}\,^{2} \,dt} = a + \frac{3}{4}\left( {m^{2} b + n^{2} c} \right).$$

Integration is performed over the prescribed interval $0 \to 2\pi /\Lambda_{1}$.

According to the ICs specified in Eq. (30), the resulting expression for Eq. (27) can be obtained as follows:

35 $$\,\,\eta_{eqv2} = \int\limits_{0}^{{2\pi /\Lambda_{2} }} {\dot{\tilde{v}}\,Q_{2} (\dot{\tilde{u}},\dot{\tilde{v}})} dt/\int\limits_{0}^{{2\pi /\Lambda_{2} }} {\dot{\tilde{v}}^{2} \,dt} = d,$$

and

36 $$\phi_{eqv2}^{2} = \int\limits_{0}^{{2\pi /\Lambda_{2} }} {\tilde{v}\,R_{2} (\tilde{u},\tilde{v})} dt/\int\limits_{0}^{{2\pi /\Lambda_{2} }} {\tilde{v}^{2} \,dt} = a + \frac{3}{4}\left( {n^{2} b + m^{2} c} \right).$$

Integration is performed over the prescribed interval $0 \to 2\pi /\Lambda_{2}$.

Accordingly, Eqs. (31) and (32) are transformed via the standard normal form through the coordinate transformations:

37 $$u(t) = \tilde{u}\,{\mathrm{Exp}}( - \eta_{eqv1} \,t/2) ,$$

and

38 $$v(t) = \tilde{v}\,{\mathrm{Exp}}( - \eta_{eqv2} \,t/2),$$

Accordingly, the governing linear ODEs may be represented as follows:

39 $$\ddot{\tilde{u}} + \Lambda_{1}^{2} \tilde{u} = 0,$$

and

40 $$\ddot{\tilde{v}} + \Lambda_{2}^{2} \tilde{v} = 0.$$

As a consequence, one gets

41 $$\Lambda_{1}^{2} = \phi_{eqv1}^{2} - \frac{1}{4}\eta_{eqv1}^{2} ,$$

and

42 $$\Lambda_{2}^{2} = \phi_{eqv2}^{2} - \frac{1}{4}\eta_{eqv2}^{2} .$$

Accordingly, the stability boundaries are regarded as:

43 $$\Lambda_{1}^{2} > 0, \; \& \,\eta_{eqv1} > 0,$$

and

44 $$\Lambda_{2}^{2} > 0, \; \mathrm{and} \; \eta_{eqv2} > 0.$$

## Guaranteeing outcomes (Case 2)

The following section is devoted to verifying the theoretical developments obtained for Case 2 (coupled quartic oscillators) by means of direct quantitative and graphical comparisons between the original nonlinear system and its NPA-based equivalent formulation.

### Tabular comparison of validation

Tables 3 and 4 provide a direct quantitative comparison between the nonlinear NS of Eqs. (23) and (24), obtained employing the MS via the command *NDSolve*, and the corresponding analytical approximations derived via the NPA from Eqs. (31) and (32). All simulations are performed over the same time interval and are initialized using identical ICs, ensuring a consistent and unbiased validation.

**Table 3:** Shows evaluation of nonlinear NS versus analytical NPA solutions for Eqs. (23) and (31) across selected time intervals.

Time	NDSolve (NS)	Analytical (NPA)	Absolute error
0	0.07	0.07	0.00
5	− 0.0165395	− 0.0165845	0.0000450071
10	− 0.0142936	− 0.0141363	0.000157244
15	0.016028	0.0160017	0.0000262846
20	− 0.00586397	− 0.00600654	0.000142577
25	− 0.00182897	− 0.00167995	0.000149016
30	0.00342607	0.00338625	0.0000398179
35	− 0.0017035	− 0.00175357	0.0000500729
40	− 0.000078881	− 0.000017637	0.0000612448
45	0.00068139	0.000657466	0.0000239241
50	− 0.000443532	− 0.000453976	0.0000104438

**Table 4:** Shows evaluation of nonlinear NS versus analytical NPA solutions for Eqs. (24) and (32) across selected time intervals.

Time	NDSolve (NS)	Analytical (NPA)	Absolute error
0	0.02	0.02	0.00
5	− 0.00472557	− 0.00473843	0.0000128592
10	− 0.00408388	− 0.00403896	0.0000449267
15	0.00457942	0.00457191	0.00000750988
20	− 0.00167542	− 0.00171616	0.0000407362
25	− 0.000522562	− 0.000479986	0.000042576
30	0.000978878	0.000967501	0.0000113765
35	− 0.000486713	− 0.00050102	0.0000143065
40	− 0.0000225377	− 0.00000503915	0.0000174985
45	0.000194683	0.000187847	0.00000683547
50	− 0.000126723	− 0.000129707	0.00000298395

For the first oscillator (Table 3), the numerical and analytical responses remain nearly indistinguishable throughout the entire time span. The maximum absolute error does not exceed $1.57 \times 10^{ - 4}$, demonstrating that the NPA-based equivalent linear ODEs accurately reproduce the nonlinear numerical conduct with high fidelity. Physically, this demonstrates that the analytical model effectively reproduces the energy exchange, nonlinear amplitude modulation, and dissipative decay inherent in the first oscillator’s dynamics. A comparable level of agreement is observed for the second oscillator (Table 4), where the maximum deviation remains below $4.5 \times 10^{ - 5}$. The small and smoothly varying error values indicate that the analytical approximation reliably tracks the nonlinear NS without any noticeable accumulation of discrepancies. This reflects the NPA’s ability to faithfully reproduce inter-modal coupling, phase relations, and subtle nonlinear interactions, even for a lower-amplitude oscillator. Together, these results highlight that the NPA provides not only computational efficiency but also a physically meaningful representation of strongly nonlinear 2DOF systems, making it suitable for practical engineering applications such as vibration control, energy transfer in mechanical oscillators, and modeling coupled micro- or nano-scale resonators. The dimensionless factor values associated with Tables 3 and 4 are: $a = 0.2,\,\,b = 0.3,\,A = 0.07,\,B = 0.02,c = 0.3,\,\,{\mathrm{and}}\,\,d = 0.2.$

### Schematic-based validation

**Fig. 9:**
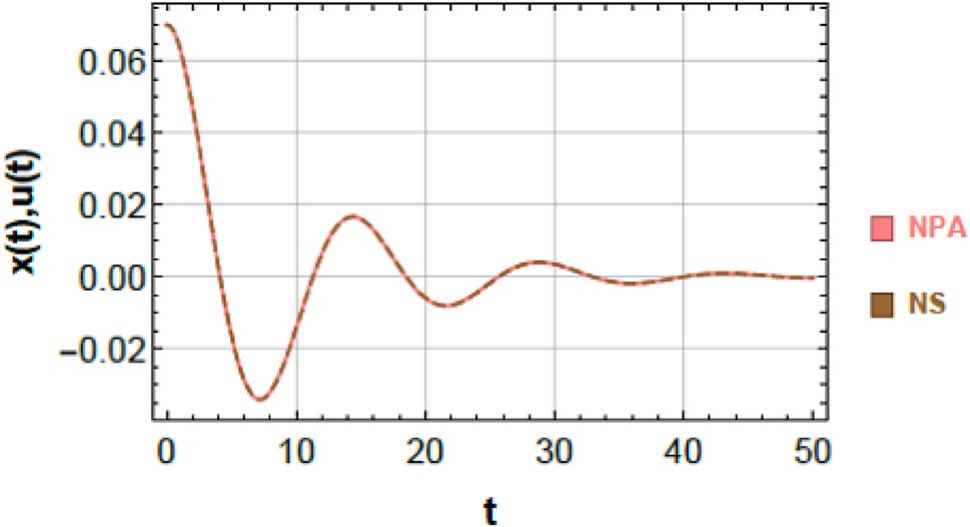
Displays a comparison of the nonlinear NS of Eq. (23) with the corresponding approximate NPA solution of Eq. (31).

**Fig. 10:**
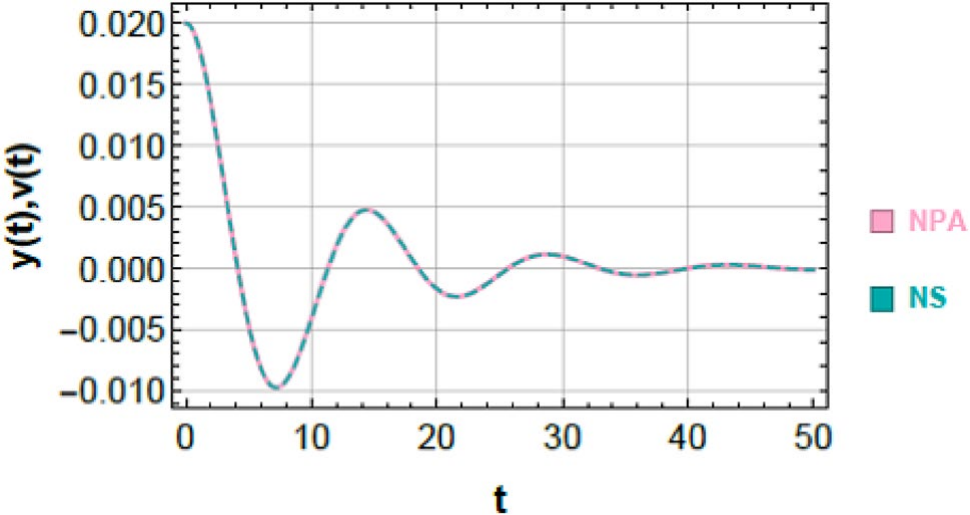
Shows a comparison of the nonlinear (NS) of Eq. (24) and the corresponding approximate NPA solution of Eq. (32).

Figures 9 and 10 present a graphical comparison between the nonlinear numerical solutions of Eqs. (23) and (24) and the corresponding NPA-based analytical solutions of Eqs. (31) and (32).

In Fig. 9, the NS of Eq. (23) (brown curve) and the analytical NPA solution of Eq. (31) (pink curve) exhibit an almost complete overlap over the entire time domain. The maximum absolute deviation is limited to $1.96 \times 10^{ - 4}$, confirming the high accuracy of the proposed equivalent formulation. Physically, this indicates that the analytical approximation accurately captures the transient nonlinear oscillations, amplitude modulation, and dissipative decay of the first oscillator. The near-perfect curve overlap demonstrates that the essential energy transfer and phase evolution within the coupled system are faithfully preserved.

Similarly, Fig. 10 compares the numerical response of Eq. (24) (cyan curve) with the analytical NPA approximation of Eq. (32) (light pink curve). The two curves remain nearly coincident, with a maximum absolute error of $5.61 \times 10^{ - 5}$. The consistency in amplitude decay, waveform shape, and phase evolution ensures the robustness of the analytical approximation for the 2DOF, even under lower-amplitude oscillations and nonlinear coupling influences.

The close correspondence observed in both figures demonstrates that the NPA-based equivalent system faithfully reproduces the dynamics of the original nonlinear model, offering a computationally efficient and analytically tractable alternative to full numerical integration. From a practical perspective, this reliability makes the NPA approach suitable for engineering applications such as coupled vibration absorbers, nonlinear energy sinks, and micro-nano-mechanical resonators, where capturing both short-term transients and long-term performance with high accuracy is essential. The assigned quantities to these dimensionless factors are taken as:$$a = 0.2,$$$$b = 0.3,\,A = 0.07,\,B = 0.02$$ $$,c = 0.3,\,\,{\mathrm{and}}\,\,d = 0.2.$$

### Bifurcation analysis (Case 2)

**Fig. 11:**
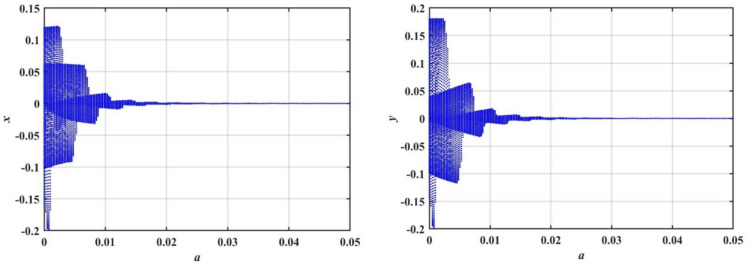
Displays a bifurcation diagram of $x$ and $y$ versus $a$.

In this part of the study, we will explore the bifurcation analysis of the 2DOF coupled quartic oscillators, as we did in the previous model. The bifurcation diagrams provided in Fig. 11 illustrate the behavior of the system’s variables $x$ and $y$, as the bifurcation parameter $a$ is varied within the range $0 \le a \le 0.05$. These diagrams highlight how the system transitions through different dynamical regimes. At the lower range of $a$, specifically when $0 \le a \le 0.02$, the distribution of points in the bifurcation diagram is dense and unbalanced. This indicates the presence of complex and chaotic behavior, characterized by the loss of periodicity and unpredictable dynamics.

**Fig. 12:**
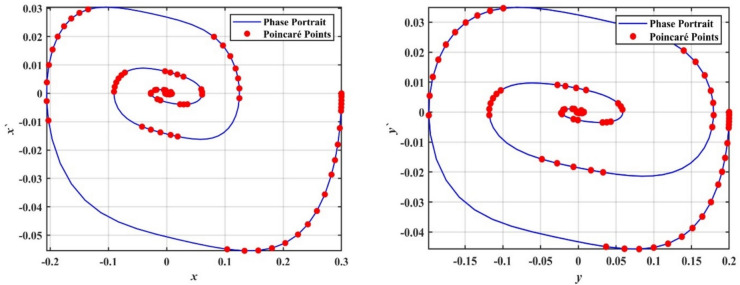
Displays a phase portrait and PMs at the chaotic state when $a = 0.005$.

**Fig. 13:**
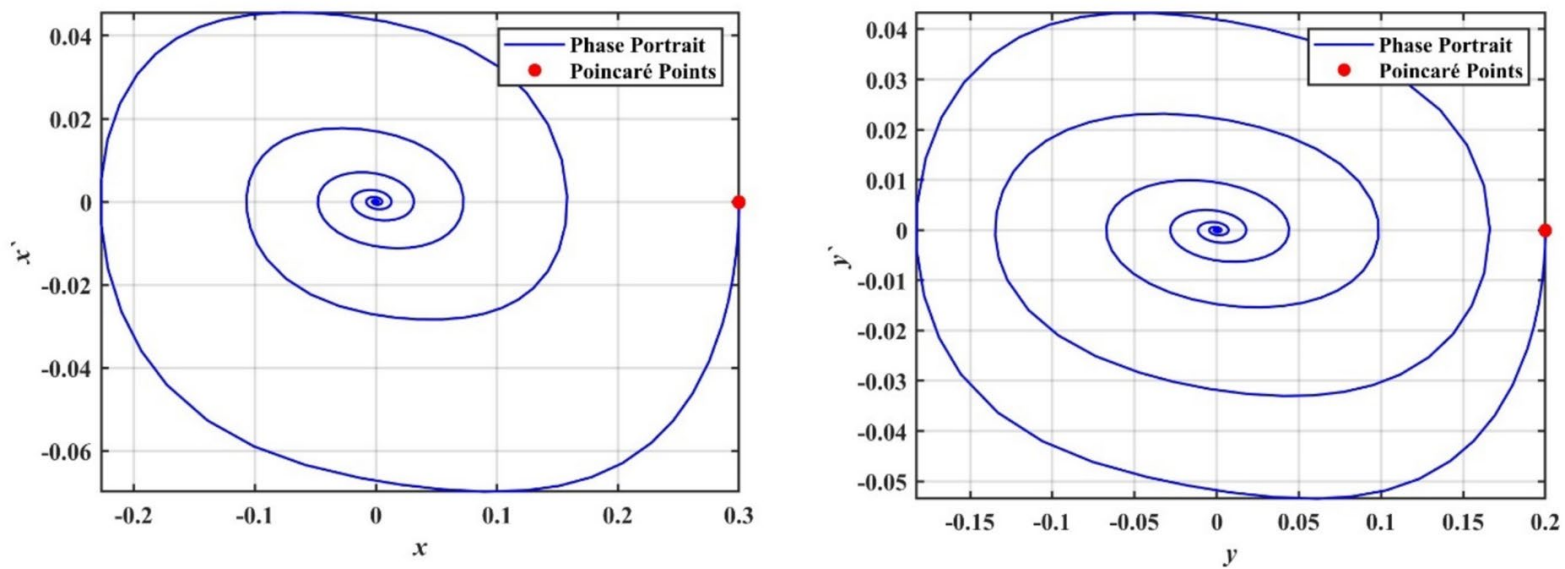
Displays a phase portrait and PMs at the periodic state when $a = 0.03$.

To further confirm this chaotic motion, phase portraits and PMs are shown in Fig. 12, supporting the observation of chaotic behavior in this region. On the other hand, as the value of $a$ increases beyond $a > 0.02$, the system appears to settle into a steady state. The points in the bifurcation diagram converge into a narrow band, suggesting that the system transitions back to a regular periodic or stable motion. This transition is corroborated by the PMs in Fig. 13, where the red points form a more predictable, organized pattern.

It is worth noting that transient responses are discarded before constructing the Poincaré maps, while the phase portraits illustrate the system’s global evolution toward the attractor, and the Poincaré sections characterize the steady-state dynamics.

### Stability assessment (Case 2)

**Fig. 14:**
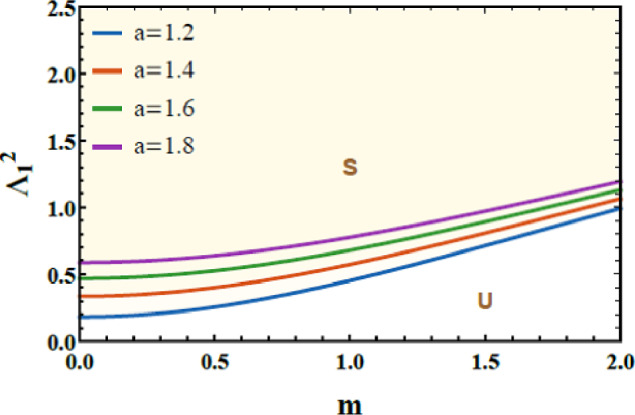
Demonstrates how $a$ affects the stability profile.

**Fig. 15:**
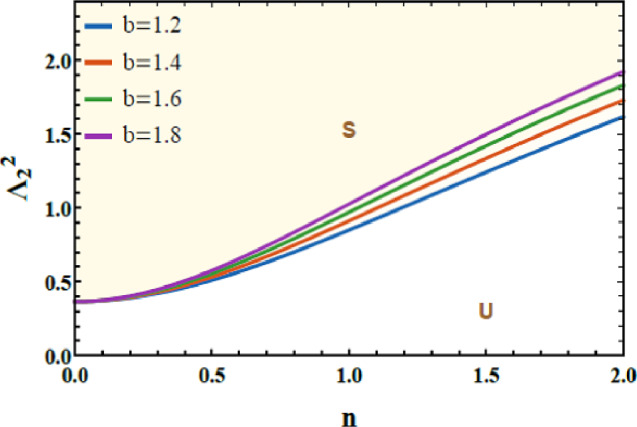
Depiction of the impact of $b$ on the stability zone.

Figures 14 and 15 are presented to evaluate the stability characteristics of the coupled quartic oscillator system of 2DOF. These figures illustrate the variation of the total frequency as a function of the initial amplitude. The imposed constraint $\Lambda_{1}^{2} > 0$ as well as $\Lambda_{2}^{2} > 0$ allowing allowing a systematic evaluation of the system response for diverse combinations of the nonlinear parameters a and b.

Figure 14 highlights the influence of parameter a, which governs the strength of the intrinsic quartic nonlinearity of the primary subsystem. As a increases from 1.2 to 1.8, the stable region within the stability map progressively contracts. From a physical standpoint, larger values of $a$ enhance the stiffness nonlinearity, intensifying internal resonance interactions and amplifying energy exchange between the coupled modes. This elevated nonlinear coupling promotes earlier loss of stability, thereby reducing the range of admissible stable oscillatory amplitudes.

Figure 15 examines the role of the parameter $b$, associated with the nonlinear coupling intensity between the 2DOF. Increasing $b$ from 1.2 to 4.2 leads to a pronounced shrinkage of the stable zones. Physically, stronger coupling accelerates energy transfer across the system, which, beyond a critical threshold, triggers nonlinear instabilities and complex dynamic transitions. This behavior confirms that excessive nonlinear coupling can be detrimental to global stability, despite its potential benefits in targeted energy redistribution. The dimensionless factors considered for these two graphs are given below as follows:

$$a = 0.2,\,\,b = 0.3,\,A = 0.07,\,B = 0.02,c = 0.3,\,\,{\mathrm{and}}\,\,d = 0.2.$$

From an engineering perspective, these results carry important implications for the design of nonlinear vibration absorbers, energy sinks, and adaptive resonator systems. The observed contraction of stability domains provides clear quantitative guidelines for selecting nonlinear stiffness and coupling parameters to ensure robust and predictable performance in mechanical and structural applications.

Moreover, the findings are directly relevant to biofluid and electro-biofluid systems, where nonlinear oscillatory mechanisms arise naturally due to fluid–structure interaction, viscoelastic effects, and Electrohydrodynamics forcing. Examples include pulsatile blood flow in compliant vessels, targeted drug delivery systems using oscillatory micro carriers, and soft robotic actuators operating in fluidic environments. In such systems, excessive nonlinear stiffness or coupling may induce undesirable oscillatory instabilities, potentially compromising functional reliability or physiological safety.

Finally, the stability maps presented in Figs. 14 and 15 offer valuable physical insight into how nonlinear parameters govern dynamic robustness. They also provide a practical framework for parameter tuning in real-world engineering, biomedical, and biofluidic applications where controlled nonlinear oscillations are essential.

## Couple simple harmonic oscillators (Case 3)

This case investigates the synchronization and phase dynamics of two coupled pendulums connected by a weak spring. When an external torque is applied to one pendulum, the system exhibits a variety of oscillatory behaviors, ranging from in-phase to out-of-phase motions, strongly influenced by the coupling strength, damping, and ICs .

In physical terms, the observed dynamics are a direct consequence of energy exchange between the two oscillators through the coupling spring. When one pendulum moves, it exerts a restoring force on the second pendulum, inducing a reactive motion. Depending on the system parameters, this leads to either synchronized oscillations (both pendulums moving in-phase) or anti-synchronized oscillations (phase-shifted motion). Damping dissipates energy from the system, gradually stabilizing the motion, while the coupling strength determines the efficiency of energy transfer and the resulting phase relationship. These phenomena illustrate fundamental concepts of nonlinear energy transfer, resonance, and synchronization in coupled oscillatory systems.

Unlike Cases 1 and 2:                                                                                                                                                                                                                                       

 • Nonlinearity is minimal, primarily arising from initial amplitude differences and weak coupling, allowing exploration of phase dynamics, synchronization patterns, and stability under damping.                                                                         • The NPA framework successfully predicts synchronous and asynchronous oscillations, amplitude modulation, and stability boundaries, demonstrating its applicability across both strongly and weakly nonlinear regimes.                                 • Stability diagrams highlight the stabilizing role of damping parameters, providing design insight for real-world applications such as vibration control, coupled resonators, and bio-inspired oscillatory networks.                                                                                                                                                                                                                                                                                                                                                                                                                                            From an engineering and practical standpoint, such a systems model:

- Mechanical vibration absorbers and tuned mass dampers.
- Coupled pendulum arrays in precision timing devices.

From a biophysical perspective, coupled oscillators provide insight into:

- Biliary and flagella motion in biological fluids.
- Cardiac pacemaker cell synchronization.
- Neural oscillations and coupled rhythmic behaviors in physiology.

Applications and real-world significance:

- Mechanical: Tuned vibration absorbers, multi-pendulum energy harvesters, MEMS devices.
- Biological: Cardiac pacemaker synchronization, biliary beating coordination, neural rhythms.
- Engineering design: Predicting phase-locked operation or preventing destructive resonance in coupled structures.

### Governing equations

The dynamics of the coupled pendulums are governed by the following nonlinear differential equations^27^:

45 $$\ddot{\phi } + \mu_{1} \dot{\phi } + \alpha_{1} \sin \phi + \beta_{1} \left( {\sin \phi - \sin \theta } \right)\cos \phi = 0,$$

and

46 $$\ddot{\theta } + \mu_{2} \dot{\theta } + \alpha_{2} \sin \theta + \beta_{2} \left( {\sin \phi - \sin \theta } \right)\cos \theta = 0.$$

### Reformulation using NPA

Applying the NPA methodology, the governing equations can be expressed in terms of equivalent linearized forms while retaining dominant nonlinear impacts:

47 $$\ddot{\phi } + H_{1} (\dot{\phi }) + K_{1} (\phi ,\,\theta ) = 0 ,$$

and

48 $$\ddot{y} + H_{2} (\dot{\theta }) + K_{2} (\phi ,\,\theta ) = 0,$$

where

49 $$\left. {\begin{array}{*{20}c} {H_{1} (\dot{\phi }) = \mu_{1} \dot{\phi }} \\ {\begin{array}{*{20}c} {K_{1} (\phi ,\,\theta ) = \alpha_{1} \sin \phi + \beta_{1} \left( {\sin \phi - \sin \theta } \right)\cos \phi } \\ {H_{2} (\dot{\theta }) = \mu_{2} \dot{\theta }} \\ {K_{2} (\phi ,\,\theta ) = \alpha_{2} \sin \theta + \beta_{2} \left( {\sin \phi - \sin \theta } \right)\cos \theta } \\ \end{array} } \\ \end{array} } \right\} .$$

The postulated solutions for Eq. (45) are presupposed in the form.

50 $$\phi = h\,\cos \omega_{1} t,\,\,\,\,{\mathrm{and}}\,\,\,\theta = {\mathrm{z}}\,\,{\text{cos }}\omega_{1} t$$

Similarly, for Eq. (46), the prescribed solutions are presupposed as:

51 $$\phi = h\cos \omega_{{2}} t,\,\,\,\,{\mathrm{and}}\,\,\,\theta = {\mathrm{z}}\,\,{\text{cos }}\omega_{{2}} t$$

These presumed responses satisfy the ICs:

52 $$\phi \left( 0 \right) = h,\,\,\dot{\phi }\left( 0 \right) = 0,\,\,\theta \left( 0 \right) = z,\,\,\,{\mathrm{and}}\,\,\,\dot{\theta }\left( 0 \right) = 0,$$

where $h,\,\,{\mathrm{and}}\,\,z$ being the initial amplitudes, and $\omega_{1,\,2}$ are the overall frequencies.

Following the previous steps in case 1, the resulting linearized ODEs take forms equivalent to Eqs. (45) and (46).

53 $$\ddot{u} + \lambda_{eqv1} \dot{u} + \Gamma_{eqv1}^{2} \,u = 0,$$

and

54 $$\ddot{v} + \lambda_{eqv2} \dot{v} + \Gamma_{eqv2}^{2} \,v = 0.$$

### Analytical evaluation

By employing the MS, the equivalent factors are computed as^18–20^:

55 $$\lambda_{eqv1} = \int\limits_{0}^{{2\pi /\omega_{1} }} {\dot{\tilde{u}}H_{1} (\dot{\tilde{u}},\dot{\tilde{v}})} dt/\int\limits_{0}^{{2\pi /\omega_{1} }} {\dot{\tilde{u}}^{2} \,dt} = \mu_{1} ,$$

and

56 $$\Gamma_{eqv1}^{2} = \int\limits_{0}^{{2\pi /\omega_{1} }} {\tilde{u}\,K_{1} (\tilde{u},\tilde{v})} dt/\int\limits_{0}^{{2\pi /\omega_{1} }} {\tilde{u}\,^{2} \,dt} = \frac{{2\alpha_{1} }}{h}J_{1} \left( h \right) + \frac{{\beta_{1} }}{h}\left( {J_{1} \left( {2h} \right) + J_{1} \left( {h - z} \right) - J_{1} \left( {h + z} \right)} \right).$$

Integration is performed over the prescribed interval $0 \to 2\pi /\omega_{1}$. In this expression, $J_{1} \left( * \right)$ represents the Bessel function, * refers to $h$ or $z$.

According to the ICs specified in Eq. (52), the resulting expression for Eq. (49) can be obtained as follows^18–20^:

57 $$\,\,\lambda_{eqv2} = \int\limits_{0}^{{2\pi /\omega_{2} }} {\dot{\tilde{v}}\,H_{2} (\dot{\tilde{u}},\dot{\tilde{v}})} dt/\int\limits_{0}^{{2\pi /\omega_{2} }} {\dot{\tilde{v}}^{2} \,dt} = \mu_{2} ,$$

and

58 $$\Gamma_{eqv2}^{2} = \int\limits_{0}^{{2\pi /\omega_{2} }} {\tilde{v}\,K_{2} (\tilde{u},\tilde{v})} dt/\int\limits_{0}^{{2\pi /\omega_{2} }} {\tilde{v}^{2} \,dt} = \frac{{2\alpha_{2} }}{z}J_{1} \left( z \right) + \frac{{\beta_{2} }}{s}\left( {J_{1} \left( {h - z} \right) - J_{1} \left( {2z} \right) + J_{1} \left( {h + z} \right)} \right).$$

Integration is performed over the prescribed interval $0 \to 2\pi /\omega_{2}$.

Accordingly, Eqs. (53) and (54) are transformed into the standard normal form through the coordinate transformations:

59 $$u(t) = \tilde{u}\,{\mathrm{Exp}}( - \lambda_{eqv1} \,t/2)$$

and

60 $$v(t) = \tilde{v}\,{\mathrm{Exp}}( - \lambda_{eqv2} \,t/2)$$

Accordingly, the governing linear ODEs reduce to

61 $$\ddot{\tilde{u}} + \omega_{1}^{2} \tilde{u} = 0$$

and

62 $$\ddot{\tilde{v}} + \omega_{2}^{2} \tilde{v} = 0.$$

As a consequence, one gets

63 $$\omega_{1}^{2} = \Gamma_{eqv1}^{2} - \frac{1}{4}\lambda_{eqv1}^{2} ,$$

and

64 $$\omega_{2}^{2} = \Gamma_{eqv2}^{2} - \frac{1}{4}\lambda_{eqv2}^{2} .$$

Accordingly, the stability constraints are expressed as:

65 $$\omega_{1}^{2} > 0, \, \& \, \lambda_{eqv1} > 0,$$

and

66 $$\omega_{2}^{2} > 0, \, \mathrm{and} \, \lambda_{eqv2} > 0.$$

## Confirming the outcomes (Case 3)

The reliability of the analytical formulation developed for the coupled simple harmonic oscillators is assessed using both numerical simulations and comparative evaluations, as summarized in Tables 5 and 6. These comparisons validate the accuracy of the NPA framework in reproducing the system’s dynamics over a wide temporal range.

**Table 5:** Shows a quantitative comparison between the NS and the NPA predictions for Eqs. (45) and (53).

Time	NDSolve (NS)	Analytical (NPA)	Absolute error
0	0.1	0.1	0.00
5	0.176018	0.0138048	0.162213
10	0.107067	− 0.067178	0.174245
15	0.105019	− 0.0257423	0.130761
20	0.0834296	0.041203	0.0422266
25	0.0702975	0.0279624	0.0423352
30	0.059202	− 0.0223695	0.0815715
35	0.0487934	− 0.0250373	0.0738307
40	0.0410243	0.00979507	0.0312292
45	0.0338957	0.0199785	0.0139173
50	0.0282552	− 0.0021398	0.030395

**Table 6:** Shows a quantitative comparison between the NS and the NPA predictions for Eqs. (46) and (54).

Time	NDSolve (NS)	Analytical (NPA)	Absolute error
0	0.2	0.2	0.00
5	− 0.056941	− 0.102704	0.0457626
10	− 0.0614663	0.0214236	0.0828899
15	0.0402808	0.017966	0.0223148
20	0.0105963	− 0.0244996	0.0350959
25	− 0.0182317	0.0160525	0.0342842
30	0.00172334	− 0.00583543	0.00755877
35	0.00620242	− 0.000507667	0.00671008
40	− 0.00255922	0.00261629	0.00517551
45	− 0.00149549	− 0.00223322	0.000737724
50	0.00140812	0.00110321	0.000304909

### Comparative evaluation using tabulated results

Tables 5 and 6 present a quantitative comparison between the NS of Eqs. (45) and (46) and the corresponding analytical predictions obtained from the NPA-based reduced forms in Eqs. (53) and (54).

First oscillator (Table 5): The NPA successfully captures the essential time evolution. Early-time deviations are observable due to initial transients; however, the absolute error decreases significantly as the oscillations settle into periodic motion. Residual errors remain on the order of $10^{ - 3} \, - \,10^{ - 2}$, confirming rapid stabilization and robust convergence even in the presence of weak coupling influences. In physical terms, this role corresponds to the damping-mediated energy dissipation, where the oscillator settles into a predictable amplitude and frequency regime. The NPA successfully reproduces transient energy exchanges and long-term oscillatory decay, confirming high-fidelity representation of the oscillator’s dynamics.

Second Oscillator (Table 6): The correspondence between the nonlinear NS and the NPA analytical prediction is strong. The absolute error is initially higher during early transients (e.g., $0.0829$ at $t = 10$), reflecting the system’s initial adjustment, but decreases steadily over time, dropping to just $0.0003$ at $t = 50$. This trend indicates that the NPA accurately captures the settling dynamics and the long-term oscillatory performance of the second oscillator. From a physical perspective, the second oscillator mirrors energy exchange and phase interactions with the first, demonstrating that the NPA faithfully reproduces coupled harmonic dynamics, including damping influences, amplitude decay, and synchronization tendencies.

Physical insights across both oscillators:

- Early-time errors correspond to transient energy redistribution and initial phase adjustments.
- Later-time minimal errors indicate steady-state oscillations where amplitude decay, synchronization, and damping effects are fully captured.
- This confirms that the NPA is a computationally efficient and physically consistent tool for modeling weakly coupled oscillatory systems.

Engineering Relevance: These findings are directly applicable to mechanical and structural oscillators, pendulum-based devices, MEMS/NEMS resonators, vibration absorbers, and energy transfer systems, where accurate prediction of both transient and steady-state coupled dynamics is essential for design, control, and stability analysis.

The specific numerical dimensionless parameters employed in this framework are:

Table 5: $$\alpha_{1} = 0.5,\,\alpha_{2} = 1.8,\,\,\beta_{1} = 0.01,\,\beta_{2} = 0.2,\,A = 0.4,\,\,\,B = 0.1,\,\mu_{1} = 0.3,\,\,{\mathrm{and}}\,\,\mu_{2} = 0.07.$$

Table 6: $$\alpha_{1} = 0.7,\,\alpha_{2} = 2.8,\,\,\beta_{1} = 0.1,\,\beta_{2} = 0.07,\,A = 0.2,\,\,\,B = 0.4,\,\mu_{1} = 0.2,\,\,{\mathrm{and}}\,\,\mu_{2} = 0.5.$$

### Validation through schematic diagrams

**Fig. 16:**
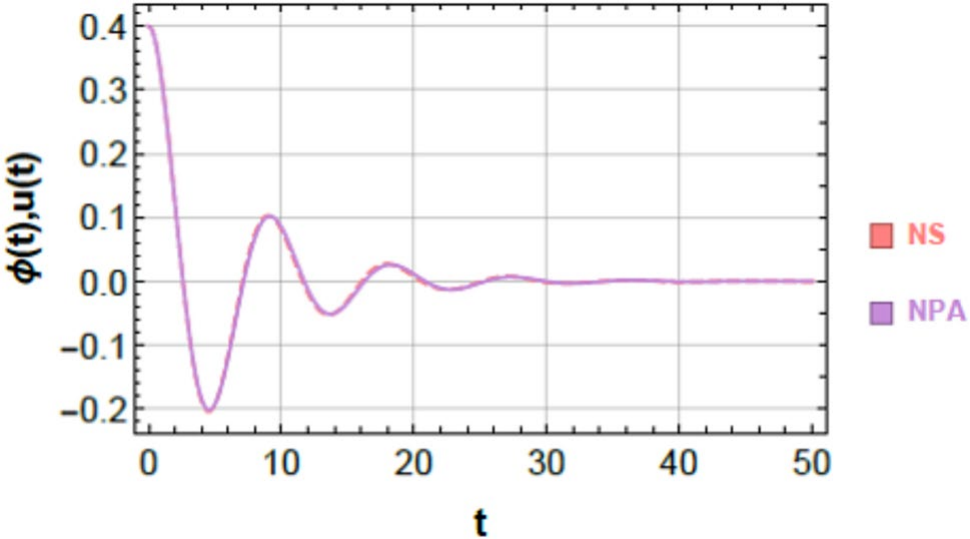
Comparison between the NS of Eq. (45) and its approximate NPA solution from Eq. (53). The amounts of the dimensionless factors are: $\alpha_{1} = 0.5,\,\alpha_{2} = 1.8,\,\,\beta_{1} = 0.01,\,\beta_{2} = 0.2,\,A = 0.4,\,\,\,B = 0.1,\,\mu_{1} = 0.3,\,\,{\mathrm{and}}\,\,\mu_{2} = 0.07.$

**Fig. 17:**
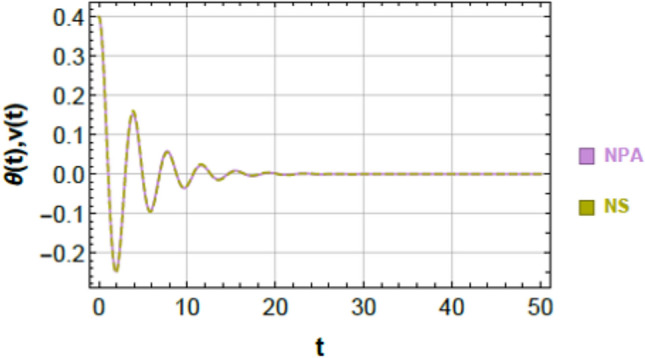
Comparison between the NS of Eq. (46) and its approximate NPA solution from Eq. (54). The amounts of the dimensionless factors are: $\alpha_{1} = 0.7,\,\alpha_{2} = 2.8,\,\,\beta_{1} = 0.1,\,\beta_{2} = 0.07,\,A = 0.2,\,\,\,B = 0.4,\,\mu_{1} = 0.2,\,\,{\mathrm{and}}\,\,\mu_{2} = 0.5.$

Figures 16 and 17 provide visual confirmation of the accuracy of the NPA approximation:

- Figure 16: Comparison between NS of Eq. (45) (pink curve) and NPA solution (purple curve) from Eq. (53). Maximum deviation: 0.00824148. The close overlap confirms that the NPA accurately reproduces temporal evolution, amplitude, and phase characteristics of the first oscillator.
- Figure 17: Comparison between NS of Eq. (46) (yellow curve) and NPA solution (purple curve) from Eq. (54). Maximum deviation: 0.00786529. The trajectories nearly coincide, illustrating the reliability of the analytical approximation for the second oscillator.

Physical interpretation and applications:

- The small residual errors demonstrate that the NPA captures both transient energy exchange and steady-state oscillatory conduct.
- Coupling strength dictates the degree of synchronization between the oscillators. Weak coupling allows slight phase differences, while stronger coupling promotes nearly in-phase motion.
- Engineering relevance: Insights apply to vibration absorbers, MEMS/NEMS devices, multi-pendulum energy harvesters, and timing mechanisms.
- Biological relevance: Models of synchronized beating of cilia or flagella, coupled neural oscillators, and cardiac pacemaker cells.

As a consequence, the NPA provides a physically consistent, computationally efficient, and analytically tractable tool for studying coupled harmonic oscillators across engineering, mechanical, and biofluid contexts.

### Bifurcation analysis (Case 3)

As with the previous two systems, we will now investigate the chaotic behavior of a pair of coupled simple harmonic oscillators. To facilitate this analysis, it is necessary to transform ODEs (44) and (45) into a system of first-order ODEs. This transformation allows us to use MATLAB to generate bifurcation diagrams, phase portraits, and Poincaré maps, which will illustrate the various types of motion exhibited by the system.

**Fig. 18:**
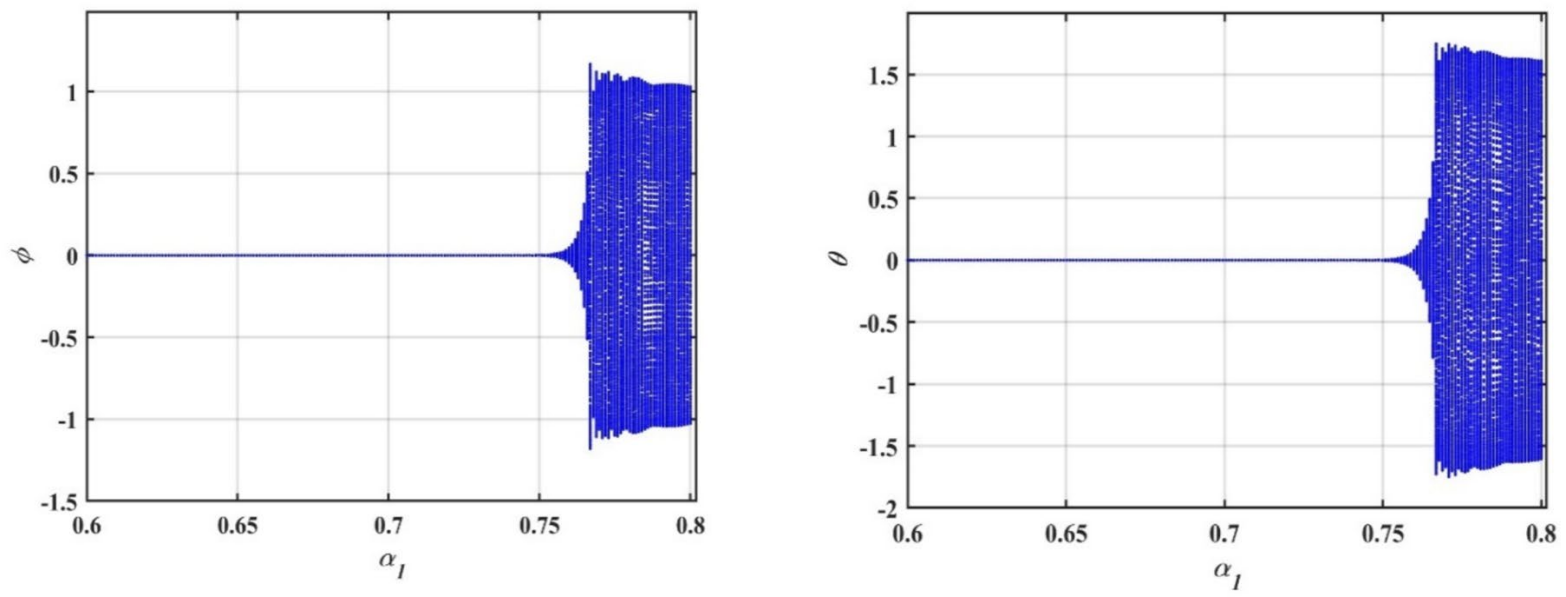
Displays bifurcation diagrams of $\phi$ and $\theta$ versus $\alpha_{1}$.

In Fig. 18, the diagrams of bifurcation for the system’s variables $\phi$ and $\theta$ clearly illustrate the transition between different dynamical behaviors as the parameter $\alpha_{1}$​ is varied. Initially, the system exhibits periodic motion for $\alpha_{1} \le 0.75$, as indicated by the orderly and repetitive structure in the bifurcation diagrams. However, as $\alpha_{1}$ exceeds 0.75, the system transitions into chaotic behavior, characterized by an irregular and dense spread of points, signifying the onset of complex and unpredictable dynamics.

**Fig. 19:**
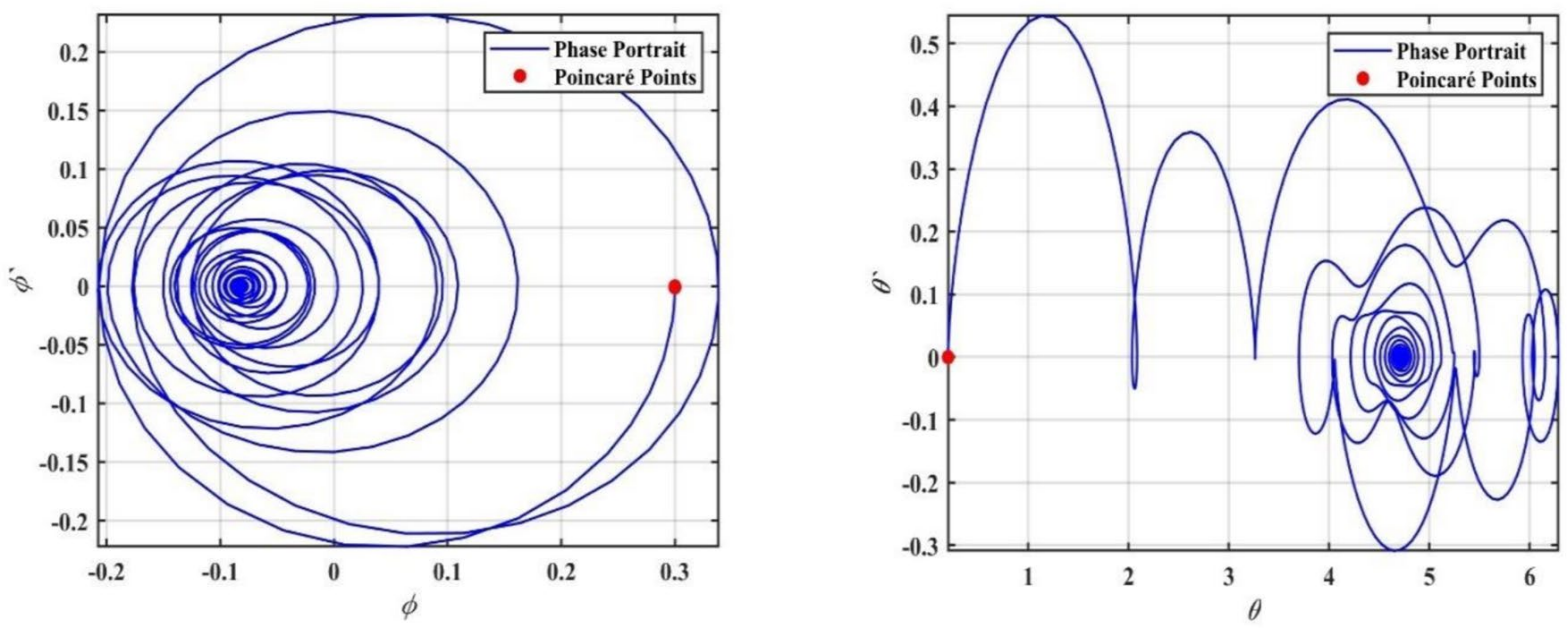
Shows a phase portrait and PMs at the periodic state when $\alpha_{1} = 0.65$.

To further confirm these transitions, phase portraits and PMs are generated for the diverse regimes of motion. In Fig. 19, the Poincaré map corresponding to the periodic motion reveals a clear and structured pattern, strongly reflecting the system’s periodic nature. This orderly distribution of points confirms the stability and regularity of the system in this regime.

**Fig. 20:**
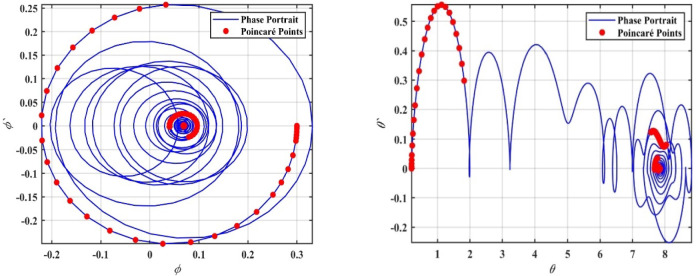
Shows a phase portrait and PMs at the chaotic state when $\alpha_{1} = 0.8$.

Conversely, the Poincaré map in Fig. 20 highlights the chaotic behavior of the system for $\alpha_{1} > 0.75$. The scattered and irregular distribution of red points in this map vividly demonstrates the loss of periodicity and the system’s transition into a chaotic state. This messy distribution serves as strong evidence of the system’s complex dynamics in this regime. By analyzing these diagrams, we have successfully identified and visualized the different types of motion in the system, reinforcing our understanding of the transitions between periodic and chaotic behaviors as influenced by the bifurcation parameter $\alpha_{1}$.

### Stability dynamics (Case 3)

**Fig. 21:**
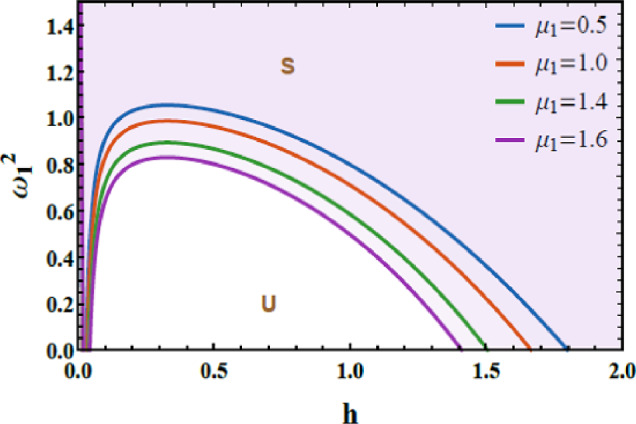
Expounds the role of $\mu_{1}$ on the stability area.

**Fig. 22:**
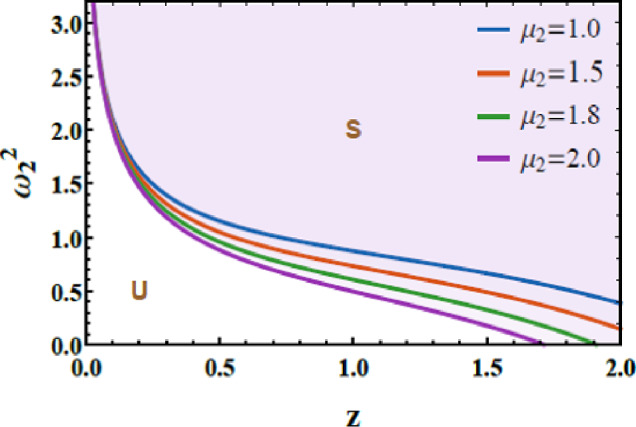
Shows the conduct of $\mu_{2}$ on the stability zone.

Figures 21 and 22 examine the stability characteristics of the coupled simple harmonic oscillator system under the constraints $\omega_{1}^{2} > 0$ as well as $\omega_{2}^{2} > 0$. These figures expound the variation of the total frequency as a function of the initial amplitudes of different combinations of the damping factors, the parameters $\mu_{1}$ and $\mu_{2}$, thereby, revealing how dissipation influences the overall system performance.

Figure 21 demonstrates the impact of varying $\mu_{1}$ on the system’s stability performance. It is observed that the stable region within the stability map expands as $\mu_{1}$ escalates from 0.5 to 1.86. This indicates that higher primary damping enhances system stability by dissipating excess energy more efficiently, thereby reducing the likelihood of resonance-induced instabilities. Physically, the damping counteracts the energy injected by initial displacements, smoothing out transient oscillations and promoting long-term regular motion.

As shown in Figure 22,increasing the secondary damping factor $\mu_{2}$​ from 1.0 to 2.0 similarly broadens the stable zones, confirming that dissipation associated with the second oscillator also provides a stabilizing influence. This effect is particularly important in coupled systems, where energy transfer between oscillators can otherwise amplify instabilities.

Physical interpretation

- The expansion of stable regions with increasing damping demonstrates the classical trade-off between energy dissipation and oscillatory amplitude.
- Properly tuned damping ensures robust synchronous behavior and mitigates the risk of chaotic or divergent responses, which is critical in engineering systems where oscillatory stability is required.

Applications

- Engineering: Design of vibration absorbers, mechanical linkages, MEMS/NEMS resonators, and precision timing devices, where controlled damping is essential to maintain performance.
- Biofluids: Modeling of coupled biological oscillators, such as biliary beating or cardiac pacemaker cells, where damping represents physiological resistance mechanisms.
- Structural systems: Bridges, tall buildings, or coupled pendulum-based energy harvesters, where controlled damping improves resilience against environmental perturbations.

The dimensionless factors adopted for this framework are:

Figure 21: $$\alpha_{1} = 0.5,\,\alpha_{2} = 1.8,\,\,\beta_{1} = 0.01,\,\beta_{2} = 0.2,\,A = 0.4,\,\,\,B = 0.1,\,\mu_{1} = 0.3,\,\,{\mathrm{and}}\,\,\mu_{2} = 0.07.$$

Figure 22: $$\alpha_{1} = 0.7,\,\alpha_{2} = 2.8,\,\,\beta_{1} = 0.1,\,\beta_{2} = 0.07,\,A = 0.2,\,\,\,B = 0.4,\,\mu_{1} = 0.2,\,\,{\mathrm{and}}\,\,\mu_{2} = 0.5.$$

## Concluding insights

The present work provides a unified analytical investigation of three distinct 2DOF oscillatory systems, ranging from weakly coupled harmonic oscillators to strongly nonlinear van der Pol–Duffing and quartic systems. By systematically examining these configurations, the study highlights the flexibility of the NPA in capturing a broad spectrum of dynamic behaviors, including synchronization, internal resonance, amplitude modulation, and chaotic motion. Importantly, the work validates that a single analytical framework can consistently handle systems with varying degrees of nonlinearity, coupling, and dissipation, bridging the gap between linear approximations and fully nonlinear numerical simulations. This integrative perspective emphasizes the novelty of the approach, showcasing its ability to provide accurate, computationally efficient, and physically interpretable solutions across multiple classes of coupled oscillatory systems.

The main methodological insights and novel contributions of this work can be summarized as follows:

1. *Case 1*: Coupled van der Pol Oscillators: Systems with amplitude-dependent self-excitation and cubic stiffness, exhibiting rich phenomena including synchronization, amplitude modulation, and bifurcations.
2. *Case 2*: Coupled Quartic Oscillators: Strongly nonlinear systems dominated by quartic restoring forces, showcasing internal resonance, nonlinear energy transfer, and complex phase-space structures.
3. *Case 3*: Coupled Simple Harmonic Oscillators: Nearly linear systems with weak coupling, allowing detailed exploration of phase dynamics, synchronization, and damping-induced stability.

Across these cases, the NPA framework was applied to derive accurate analytical approximations, capturing the essential dynamics of each system without relying on small-parameter assumptions or Taylor expansions. The methodology demonstrated:

1. *Accuracy:* Excellent agreement with full numerical simulations for both transient and long-term behaviors.
2. *Physical Interpretability:* Preservation of energy transfer, amplitude decay, mode interactions, and phase dynamics.
3. *Versatility:* Unified treatment of weakly and strongly nonlinear, self-excited, and coupled systems.
4. *Computational Efficiency:* Analytical findings obtained without the cost of extensive numerical integration.

### Novelty and contribution

The main novelty of this work lies in demonstrating that a single NPA framework can handle diverse nonlinear behaviors:

1. From weakly coupled harmonic oscillators (Case 3) to self-excited cubic systems (Case 1) and strongly nonlinear quartic oscillators (Case 2).
2. Capturing both transient dynamics and steady-state responses with high fidelity.
3. Providing a framework capable of stability assessment, amplitude-frequency prediction, and phase analysis across all cases.

### Real-world applications

The presented methodology has broad practical implications across multiple fields:

1. Mechanical and Aerospace Systems: Nonlinear vibration analysis, stabilization, and absorber design.
2. Structural Engineering: Predicting nonlinear responses of coupled components under dynamic loading.
3. MEMS/NEMS: Design of nonlinear sensors and actuators with predictable energy transfer.
4. Nonlinear Energy Management: Analysis and design of nonlinear energy sinks, coupled resonators, and vibration control devices.
5. Biological and Biofluid Systems: Modeling of coupled oscillatory networks, such as cardiac pacemaker cells, neuronal networks, or culinary dynamics, where synchronization and damping play crucial roles.

### Future extensions may include

1. Incorporation of time- or state-dependent damping, fractional, or viscoelastic effects.
2. Analysis of external and parametric excitations, including multi-frequency and modulated inputs.
3. Extension to higher-dimensional oscillator networks, lattice structures, and chains of coupled oscillators.
4. Integration with data-driven or machine-learning approaches for real-time parameter estimation, adaptive control, and hybrid analytical–computational modeling.

As a consequence, this work demonstrates that the NPA provides a unified, robust, and physically interpretable analytical framework capable of addressing a wide range of coupled nonlinear oscillatory systems, bridging the gap between theory, numerical validation, and real-world applications. The examination of delay-coupled nonlinear dynamics will be addressed^28,29^.

## Data Availability

All data generated or analyzed during this study are included in this manuscript.
